# Antibodies as biomarkers for cancer risk: a systematic review

**DOI:** 10.1093/cei/uxac030

**Published:** 2022-04-04

**Authors:** Maria J Monroy-Iglesias, Silvia Crescioli, Kerri Beckmann, Nga Le, Sophia N Karagiannis, Mieke Van Hemelrijck, Aida Santaolalla

**Affiliations:** Translational Oncology and Urology Research (TOUR), Centre for Cancer, Society, and Public Health, School of Cancer and Pharmaceutical Sciences, King’s College London, London, UK; St. John’s Institute of Dermatology, School of Basic & Medical Biosciences, King’s College London, London SE1 9RT, UK; Higher Degree by Research, University of South Australia, Adelaide, Australia; Cancer Epidemiology and Population Health Research Group, University of South Australia, Adelaide, SE, Australia; Higher Degree by Research, University of South Australia, Adelaide, Australia; St. John’s Institute of Dermatology, School of Basic & Medical Biosciences, King’s College London, London SE1 9RT, UK; Translational Oncology and Urology Research (TOUR), Centre for Cancer, Society, and Public Health, School of Cancer and Pharmaceutical Sciences, King’s College London, London, UK; Translational Oncology and Urology Research (TOUR), Centre for Cancer, Society, and Public Health, School of Cancer and Pharmaceutical Sciences, King’s College London, London, UK

**Keywords:** antibodies, biomarkers, cancer, early detection, tumor -associated antigens, immunoglobulin

## Abstract

Increasing evidence has linked the humoral immune response with the development of various cancers. Therefore, there is growing interest in investigating the predictive value of antibodies to assess overall and tissue site-specific cancer risk. Given the large amount of antibody types and the broad scope of the search (i.e. cancer risk), the primary aim of this systematic review was to present an overview of the most researched antibodies (i.e. immunoglobulin (Ig) isotypes (IgG, IgM, IgA, and IgE), tumour and self-antigen-reactive antibodies, infection-related antibodies) in relation to overall and site-specific cancer risk. We identified various antibody types that have been associated with the risk of cancer. While no significant associations were found for IgM serum levels, studies found an inconsistent association among IgE, IgA, and IgG serum levels in relation to cancer risk. When evaluating antibodies against infectious agents, most studies reported a positive link with specific cancers known to be associated with the specific agent recognized by serum antibodies (i.e. helicobacter pylori and gastric cancer, hepatitis B virus and hepatocellular carcinoma, and human papillomavirus and cervical cancer). Several reports identified autoantibodies, as single biomarkers (e.g. anti-p53, anti-MUC1, and anti-CA125) but especially in panels of multiple autoantibodies, to have potential as diagnostic biomarkers for specific cancer types. Overall, there is emerging evidence associating certain antibodies to cancer risk, especially immunoglobulin isotypes, tumour-associated antigen-specific, and self-reactive antibodies. Further experimental studies are necessary to assess the efficacy of specific antibodies as markers for the early diagnosis of cancer.

## Teaser

The humoral immune response has been consistently linked with the development of various cancers. Therefore, increasing evidence has investigated the predictive value of antibodies to assess site-specific cancer risk. In the current study, we review the current evidence for the association between the most researched antibodies and risk of site-specific cancers

## Introduction

**Figure 1:**
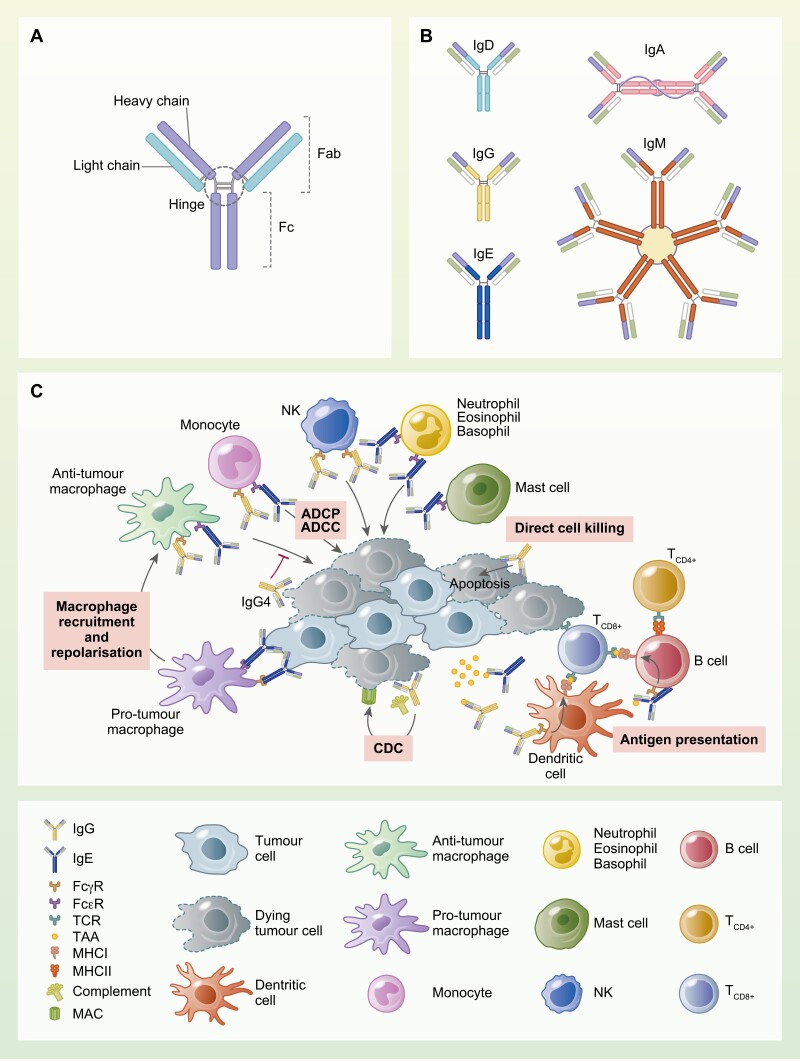
(A) Schematic representing antibody structure with heavy and light chains, and Fab, hinge, and Fc regions. (B) Heavy chain constant regions of different isotype are labelled in: light blue (IgD), yellow (IgG), blue (IgE), pink (IgA), red (IgM); IgM and IgA J chain is in blue. (C) Antibody-mediated anti-tumour or pro-tumour effector functions. Antibodies can exert several anti-tumour effector functions: mediating ADCC, ADCP, and CDC. Antibodies engaged with FcRs on immune effector cells and bound to tumour-derived antigens to form immune complexes, can (a) repolarize immune cells such as NK cells and pro-tumour macrophages into pro-inflammatory, anti-tumour phenotypes and (b) facilitate antigen internalization, processing, and presentation to activate T cells. Antibodies can also exert direct cell killing, via antigen neutralization and blocking of downstream signalling, resulting in block of tumour growth and induction of apoptosis. Some IgG subclasses, such as IgG4, can exert pro-tumour functions. IgG4 has poor capacity to fix complement and lower ability to bind activating FcγRs and therefore lower ability to trigger effector functions compared to IgG1, and relatively high affinity for the inhibitory receptor FcγRIIb, resulting in negative immune effector cell activating signals, potentially blocking IgG1 mediated effector functions. See online supplementary material for a colour version of this figure.

Immunoglobulins (Ig) are tetrameric glycoproteins produced by B cells as part of the humoral immune response. Their structure is composed of a Fab region, consisting of two identical Fab fragments, including the light chain and part of the heavy chain; a fragment crystallizable (Fc) region formed by the constant portion of the two heavy chains; and a hinge region, joining the Fab and Fc regions (Fig. 1). The heavy chain defines the isotype of the antibody, and the Fc portion can bind cognate Fc receptors (FcRs) on immune cells and members of the complement cascade including complement component 1q (C1q) and is responsible for antibody-mediated effector functions such as antibody-dependent cell cytotoxicity (ADCC), antibody-dependent cell phagocytosis (ADCP), and complement-dependent cytotoxicity (CDC) [1]. Antibodies, binding to FcRs expressed on immune cells, can also influence immune cell phenotype, and polarization and once complexed with antigens to form immune complexes, they can be internalized to facilitate antigen presentation. Human B cells can express five antibody classes (divided into nine antibody isotypes, IgD, IgM, IgG [1–4], IgA [1, 2], and IgE). Each class recognizes specific cognate FcRs or C1q with different affinity and thus differ in their abilities to trigger effector functions such as ADCC, ADCP, and CDC. Therefore, antibody isotype may significantly influence the immune response that may protect not only against external pathogens but also from the rise of cancer. The IgM isotype is involved in primary immune response and in its secreted form it can assemble in high avidity pentamers. IgG is the predominant class of antibodies in the human serum. IgG subclasses like IgG1 and IgG3 have a high affinity for activating FcγRs and C1q resulting in a high capacity to trigger ADCC and activate the complement cascade. IgG2 and IgG4 subclasses have instead poor capacity to fix complement and lower ability to bind activating FcγRs compared to IgG1, and IgG4 has a relatively high affinity for the inhibitory receptor FcγRIIb resulting in negative immune effector cell activating signals and lower ability to trigger effector functions. IgA is the predominant isotype in mucosal surfaces and in secretions, and its neutralizing capacity is crucial for protecting mucosal surfaces from toxins, viruses, and bacteria. It has a low capacity to activate complement but can engage neutrophils and trigger strong ADCC. IgE antibodies are usually associated with hypersensitivity and allergic reactions as well as responses to parasitic worm infections. IgE can trigger ADCC and ADCP as well as being able to facilitate antigen presentation and, in the context of cancer immune surveillance, being able to repolarize pro-tumour macrophages into pro-inflammatory, anti-tumour phenotypes [2]. In addition to antibody isotypes, another feature that can influence antibody effector function is antibody glycosylation, which might modulate Fc receptors’ affinity and consequently antibody effector function. This has been widely studied for IgG isotypes, with interesting findings on the effects of fucosylation, galactosylation, and sialylation [3]. Of notice, alterations in IgG galactosylation have been reported as a biomarker for multiple cancer types [4]. Antibodies can also have a direct effect by binding to the target antigen. For cell surface antigens involved in downstream signalling, antibody-target engagement can sometimes have an agonistic effect on the target which could result in activation of a signalling cascade, but most often the binding of the antibody could have an antagonistic/inhibitory effect on the target’s downstream signalling functions. This can result in impaired cell growth and apoptosis; for cell surface antigens involved in cell–cell interactions or adhesion, antibody binding could impair or prevent these processes resulting in inhibition of tumour progression (Fig. 1) [1].

It has been suggested that the humoral immune system plays an important role in both the support and suppression of carcinogenesis [5]. For instance, several studies have reported the ability of B cells to inhibit tumour development through the production of tumour-reactive antibodies [6]. However, B cells can also contribute to immune tolerance and allow tumour development by producing immunosuppressive cytokines and antibodies which are ineffective in mediating immune effector functions [6]. Moreover, the humoral immune system is crucial for protection against invading pathogens and plays a critical role in the control and suppression of malignant cells via immunosurveillance. Therefore an imbalance in the immune system homeostasis may have an effect in carcinogenesis. There is ample evidence linking prior and chronic exposure to several infectious agents with a higher risk of cancer (i.e. human papillomavirus (HPV), Epstein-Barr virus (EBV), and *Helicobacter pylori* (HP)). Moreover, epidemiological evidence has pointed to significant associations between autoimmune disorders and cancer risk. An increased risk of malignancies has been observed previously in different autoimmune disorders.

Furthermore, immunoglobulins against self-antigens and tumour-associated antigens (TAAs) have been found both in the serum of patients with cancer and in the tumour microenvironment [7, 8]. Tumours can produce TAAs either by mutational mechanisms (mutated tumour-specific antigens, mTSAs) or by non-mutational mechanisms (non-mutational TAAs, nmTAAs), which could be overexpressed in cancer compared to normal tissue or may be cancer-specific. TAAs may induce an immune response. Humoral immune surveillance mechanisms may be protective against tumour cells and inhibit cancer growth, however, if the antigens are not tumour specific, the immune system can also recognize antigen-expressing non-malignant cells resulting in autoimmune reactions [7, 9, 10]. However, the propensity of tumour cells to escape immune surveillance may be a key step in tumorigenesis [6].

The presence, specificity, and isotype distribution of Igs in patients with cancer likely have an impact on tumour progression and could potentially inform on early detection of cancer and even predict the survival of the patient [6, 11]. Procedures to test the presence of antibodies, especially serum antibodies, are minimally invasive and easy to measure, and for this they harbour potential as biomarkers for cancer. Therefore, evaluating the link between antibodies and cancer risk and validating antibodies as biomarkers for diagnostic purposes are crucial. This may be especially beneficial in relation to cancers for which screening tests are currently lacking, but for which earlier detection would provide a substantial chance to treat promptly and offers a better chance of prolonged survival. In the present study, we aimed to outline the current evidence for the associations between the most researched immunoglobulin types and the risk of tissue site-specific cancers, and for the utility of Igs as biomarkers for cancer detection.

## Methods

### Data sources and searches

The current systematic review was performed in accordance with the preferred reporting items for systematic reviews and meta-analyses (PRISMA) guidelines [12]. We performed a literature search of epidemiological studies using PubMed with the search terms presented in Table 1. We included human studies published in English between 1 January 2000 and 9 September 2021. After preliminary screening of titles and abstracts, five independent reviewers (MM, NL, KB, SC, and AS) assessed the full text and reference lists of relevant publications for final inclusion; articles cited as references that were considered to be potentially relevant were also reviewed.

**Table 1:** Search strategy followed in the search engine PubMed on 9th September 2021.

	Search terms	Hits
**Restrictions**	Humans	
	Full-text	
	English	
	Adult	
	From 01/01/2000	
**Malignancy**		
#1	“Cancer” [Title/Abstract] OR “Leukemia” [Title/Abstract] OR “Lymphoma” [Title/Abstract] OR “Myeloma” [Title/Abstract] OR “Leukaemia” [Title/Abstract] OR “Carcinoma” [Title/Abstract] OR “Neoplasm” [Title/Abstract] OR “Malignant tumor” [Title/Abstract] OR “Malignant tumour” [Title/Abstract]	605,152
#2	“Neoplasms” [Mesh]	816,183
#3	#1 AND #2	557,416
#4	“cancer risk” [Title/Abstract]	639,508
#5	“risk*” [Title/Abstract]	
#6	#4 OR #5	859,604
#7	#3 AND #6	146,542
**Immunoglobulin**		
#8	“Immunoglobulin*” [Title/Abstract] OR “Serum Immunoglobulin*” [Title/Abstract] OR “serum Ig*” [Title/Abstract] OR “Antibod*” [Title/Abstract] OR “Autoantibod*” [Title/Abstract] OR “IgG” [Title/Abstract] OR “IgE” [Title/Abstract] OR “IgM” [Title/Abstract] OR “IgA” [Title/Abstract] OR “IgD” [Title/Abstract]	136,508
#9	#7 AND #8	3,874
**Exclusions**		
#10	((“Therapeutics” [Mesh]) OR “Pharmacology” [Mesh]) OR “Therapeutic Uses” [Mesh] OR “Treatment” [Title/Abstract]	1,812,442
#11	#9 NOT #10	2,126

### Study selection

Only epidemiological studies looking at the association between any serum immunoglobulin antibodies and cancer risk were included. No publications exploring antibodies as potential markers of cancer survival or cancer prognosis were included. We also excluded publications using immunoglobulins as molecular markers in experimental studies such as imaging techniques and/or treatments.

The inclusion criteria considered studies on adults only. Single case studies were excluded. No other restrictions were placed on publication type, with all systematic reviews, narrative reviews, meta-analyses, original research articles (experimental, observational, and clinical trials), commentaries, letters, and editorials identified in the PubMed search, being considered eligible. Non-English publications, duplicate studies, preprints, errata, and animal studies were excluded. Moreover, only publications with full text available were included.

For each selected study, the following study characteristics were extracted into a designated datasheet: name of the first author, year of publication, study location, study design, number of participants, exposure, outcome (i.e. cancer type), main findings, and other observations.

## Results

**Figure 2:**
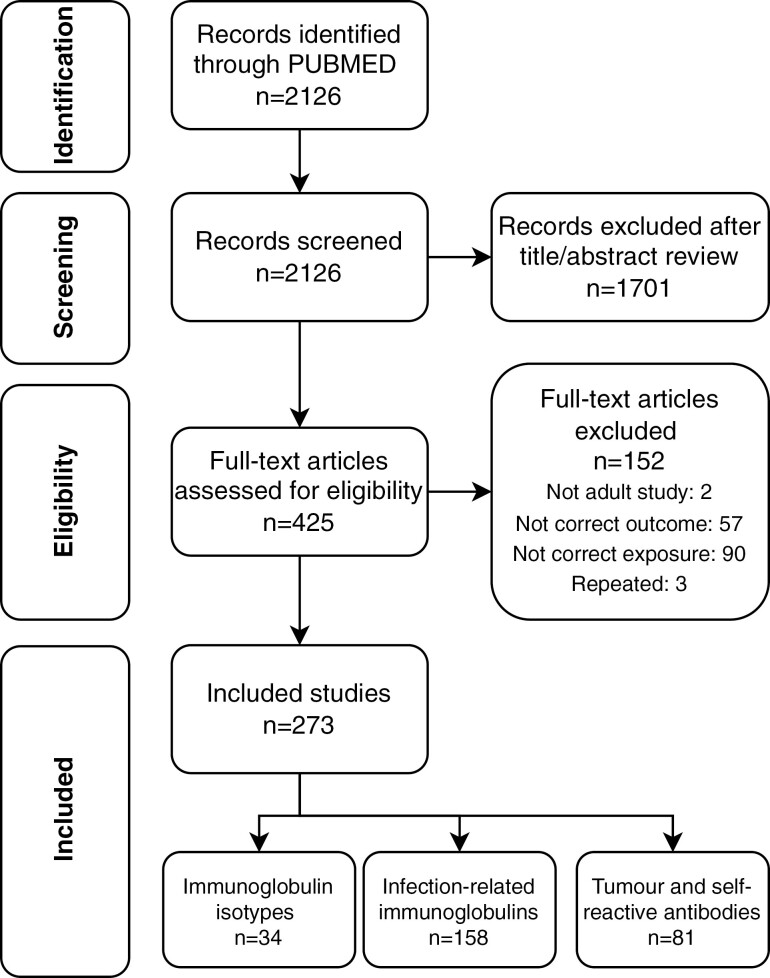
PRISMA diagram representing the systematic review strategy.

Figure 2 shows the PRISMA flowchart illustrating the study selection procedure. Our PubMed search resulted in a total of 2126 studies. A full-text review was undertaken on 425 potentially eligible articles after title and abstract screening. Following full-text review, 273 publications were included. Of the 152 full-text articles excluded, 2 were looking at paediatric populations, 56 explored a different outcome (i.e. not cancer risk), 90 investigated a different exposure (i.e. antibodies), and 3 were repeated studies. Moreover, information on publications referenced in Tables 2–6 can be found in Supplementary Table S1.

**Table 2:** Summary of results of associations between immunoglobulin isotypes and site-specific cancer risks. The strength of association is defined by the number of studies reporting on the association, the range of the hazard ratio/odds ratio/relative risks/standardized incidence ratio reported in each study, and the statistical significance.

Exposure		Population	Outcome	Number of studies	Main findings
Total IgE	IgE deficiency	General population	Overall	2	Strong negative association
	High total IgE		Overall	4	Intermediate positive association
			Pancreatic cancer	1	No significant association
			Lymphoma, leukaemia, myeloma	2	Strong positive association
			Prostate (high PSA)	1	Weak positive association
			Head and neck	1	Intermediate positive association
	High allergen-specific IgE (serum)		Skin cancer	1	No significant association
			Lung	1	No significant association
			Breast	2	No significant association
			Prostate	1	Intermediate positive association
			Lymphoma	1	No significant association
			Colon, rectum	1	No significant association
			Brain (Glioma)	1	Intermediate negative association
			Pancreatic	1	No significant association
			Skin cancer	1	Intermediate positive association
	Self-reported allergies		Overall	1	No significant association
			Lung	1	Intermediate negative association
			Breast	1	No significant association
			Prostate	1	No significant association
			Lymphoma	1 (pooled analysis of 13 studies)	Strong negative association
			Pancreatic	1	No significant association
	High asthma-specific IgE (SR)		Overall	1	No significant association
			Lung	1	Intermediate positive association
			Breast	1	No significant association
			Prostate	1	No significant association
			Lymphoma	1 (pooled analysis of 13 studies)	Strong negative association
IgA	High total IgA	General population	Overall	3	No significant association
			Pancreatic	1	No significant association
			Melanoma	1	No significant association
			Bladder	1	No significant association
			Solid cancers	1 (meta-analysis 14 studies)	Strong positive association
			Lymphoma	2	Strong negative association
			Gastrointestinal	2	Strong negative association
IgG	High total IgG	General population	Overall	1	No significant association
			Pancreatic	1	Weak negative association
			Melanoma	1	No significant association
			Bladder cancer	1	No significant association
			Solid cancers	1 (meta-analysis 14 studies)	No significant association
		Patients with IgG4 RD	Overall	2	Intermediate positive association
			Lymphoma	1	Intermediate positive association
IgM	High total IgM	General population	Overall	1	No significant association
			Pancreatic	1	No significant association
			Melanoma	1	No significant association
			Bladder	1	No significant association
			Solid cancers	1 (meta-analysis 14 studies)	No significant association
			Leukaemia	1	Intermediate positive association
	High SCCA-IgM	Patients with cirrhosis	Hepatocellular carcinoma	3	No significant association

**Figure 3:**
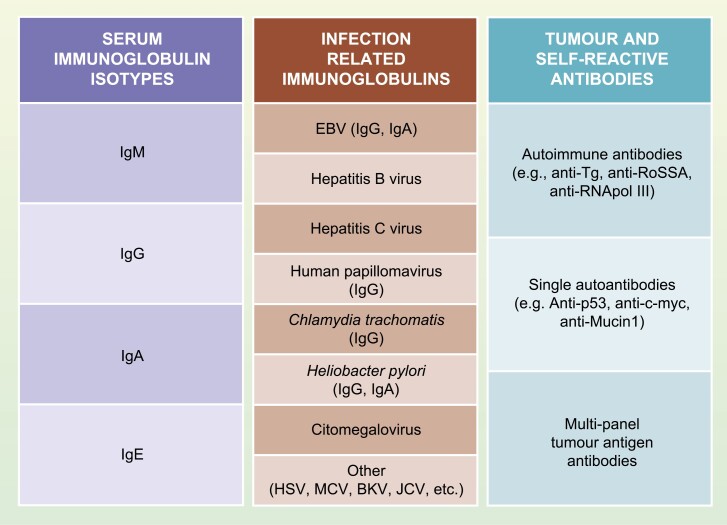
Overview of antibodies associated with cancer risk described in the review.

We observed three main categories in the publications, namely, serum immunoglobulins (*n* = 34), infectious agent-associated immunoglobulins (*n* = 158), and tumour and self-antigen reacting antibodies (*n* = 81). Therefore, the systematic review is structured following these main groupings. An overview of the main antibodies identified in the current review is illustrated in Fig. 3.

## Immunoglobulin M, G, A, and E

We identified 34 papers that assessed the risk of cancer in relation to the different immunoglobulin isotypes: IgE (*n* = 15), IgA (*n* = 7), IgG (*n* = 6), and IgM (*n* = 6). All studies followed an observational type of study design (case-control or cross-sectional designs). No clinical trials were identified. Most studies investigated the general population, except for three papers exploring IgM in patients with cirrhosis, and one exploring patients with IgG4-related diseases (IgG4-RD). An overview of the main findings is given in Table 2.

### Immunoglobulin M (IgM)

Six studies were found looking into the association between IgM levels and the risk of cancer. One study reported an increased risk of chronic lymphocytic leukaemia in patients with increased levels of IgM [13]. No associations were found with other cancer types (i.e. overall, pancreatic, melanoma, bladder, and hepatocellular carcinoma) [14–16].

### Immunoglobulin G (IgG)

Six studies have looked into the link between serum IgG levels and risk of site-specific cancer, however, not many studies explored the association with overall cancer. One cohort study found no association between serum IgG and overall cancer risk [17]. When looking at site-specific cancers, a large cohort study reported a negative association between serum IgG and risk of pancreatic cancer [16]. No associations were found for other cancer types (i.e. melanoma, bladder) [5, 14, 15]. Furthermore, a study focused on the association between IgG4-related disease (IgG4-RD), an inflammatory condition, and the risk of numerous cancer types. This observational study reported that the patients with IgG4-RD disease were at higher risk of overall cancer and lymphoma [18].

### Immunoglobulin A (IgA)

Epidemiological studies have reported an inconsistent relationship between IgA levels and the risk of cancer [19, 20]. A meta-analysis of 14 studies found a strong positive association with solid cancers [5]. However, a strong negative association was also found between IgA and the risk of gastrointestinal cancer and lymphoma [19]. Moreover, no significant associations were found in various studies looking at overall, pancreatic, melanoma, and bladder cancer risk, in relation to IgA levels [14–16].

### Immunoglobulin E (IgE)

Atopy and allergies are defined by exaggerated IgE responses to environmental allergens. We found 12 studies looking at the association between overall IgE (total concentration of IgE in serum) and the risk of various cancer types. Two cohort studies looking at total serum IgE reported a negative association with overall risk of cancer while two large cohorts found no significant associations with overall cancer risk [21, 22]. Moreover, four large cohort studies, including two with data from the European Prospective Investigation into Cancer and Nutrition (EPIC) cohort, found that IgE-deficiency and ultra-low IgE levels were strongly associated with an increased risk of overall cancer [23–25]. One of these studies reported a strong positive association between low levels of serum IgE and the risk of chronic lymphocytic leukaemia, lymphomas, and multiple myeloma [25]. On the other hand, a large case-control study reported a strong positive association between IgE and head and neck cancers [26]. No other significant associations were found with other cancer types (i.e. pancreatic, prostate) [27].

Several studies have investigated the association between self-reported allergies and allergen-specific IgE, with varying results. A cohort study reported a positive association between serum allergen-specific IgE and risk of prostate cancer [28]. Moreover, a population-based case-control study found an increased risk of squamous cell carcinoma of the skin in patients with high levels of allergen-specific IgE [29]. On the other hand, a nested case–control from the EPIC cohort found a strong negative association between allergen-specific IgE and risk of glioma [30]. No associations were found between this allergen-specific IgE and overall and other specific cancer types (i.e. lung, breast, lymphoma, colon and rectum, pancreatic) [22, 28, 31]. When looking at asthma-specific IgE, a strong positive association was found with lung cancer [32]. On the other hand, a pooled analysis of 13 case-control studies found a negative association between asthma-specific IgE or self-reported food allergies, and risk of non-Hodgkin lymphoma [33]. No associations were found with other cancers (i.e. overall, breast, prostate, and pancreatic) [32].

## Cancer-promoting infectious agents

We identified 158 studies assessing the risk of cancer in relation to different antibodies against various infectious agents. All studies followed an observational type of study design. No clinical trials were identified. The most commonly described associations were for antibodies against Epstein–Barr Virus (*n* = 25), hepatitis B virus (HBV, *n* = 15), hepatitis C virus (HCV, *n* = 14), human papillomavirus (*n* = 29), *H. pylori* (*n* = 29), and chlamydia trachomatis (*n* = 6). No consistent associations were found with other infectious agents. An overview of the main findings is given in Table 3.

**Table 3:** Summary of results of associations between infection-related immunoglobulins with site-specific cancer risks. The strength of association is defined by the number of studies reporting on the association, the range of the hazard ratio/odds ratio/relative risks/standardized incidence ratio reported in each study, and the statistical significance.

Exposure	Antigen/ Immunoglobulin	Outcome	Main findings
EBV (IgG, IgM and IgA)	VCA	Gastric cancer	Intermediate positive association
	EBNA		No significant associations
	ZEBRA		No significant associations
	EA		No significant associations
	EBNA	Nasopharyngeal carcinoma	Very strong positive association
	VCA		Very strong positive association
	EA		Weak positive association
	Gp350		Weak positive association
	VCA	Breast cancer	No significant association
	EBNA		No significant association
	VCA	Lymphoma (all)	No significant association
	EBNA		No significant association
	EA		No significant association
	VCA	Ovarian cancer	Moderately positive association
Hepatitis B virus	Anti-HBs (IgG and IgM)	Hepatocellular carcinoma	Very strong positive association
	Anti-HBc (IgM)		Strong positive association
	Anti-HBc (IgG)		Strong positive association
	Anti-HBs (IgG and IgM)	Pancreatic cancer	Strong positive association
	Anti-HBc (IgM)		Weak positive association
	Anti-HBs (IgG and IgM)	Extrahepatic bile duct cancer	Weak positive association
	Anti-HBc (IgM)	Oropharyngeal	Weak positive association
Hepatitis C virus (IgM)	Anti-HCV	Hepatocellular carcinoma	Very strong positive association
	Anti-HCV	Pancreatic cancer	No significant association
	Anti-HCV	Lymphoma (all)	No significant associations
	Anti-HCV	Renal	Weak positive association
	Anti-HCV	Cholangiocarcinoma	Weak positive association
HPV (IgG)	16	Overall cancer	No significant association
	16	Oesophageal cancer	No significant association
	18		No significant associations
	6	Oropharyngeal cancer	Strong positive association
	11		No significant associations
	16		No significant association
	18		Weak positive association
	16	Lung cancer	No significant associations
	5	Non-melanoma skin cancer	No significant associations
	6		No significant associations
	8		No significant associations
	16		Intermediate positive association
	18		Intermediate positive association
	16	Cervical cancer	Very strong positive association
	18		No significant association
	16	Prostate cancer	No significant association
	18		No significant association
	33		No significant association
	16	Anogenital cancers (anus, vulvar, vaginal, penile)	Strong positive association
	18	Anogenital cancers (anus, vulvar, vaginal, penile)	Strong positive association
C. Trachomatis (IgG)		Ovarian cancer	Strong positive association
		Cervical cancer	No significant associations
		Prostate cancer	Low negative association
*H. Pylori*	IgG	Gastric cancer	Intermediate positive association
	IgA		Strong positive association
	CagA		Strong positive association
	VacA		Low positive association
	CagA	Pancreatic cancer	Low positive association
	CagA	Colorectal carcinoma	Intermediate positive association
	Anti-*H. Pylori*	Lymphoma	No significant association
Herpes simplex virus 2 (IgG)		Prostate cancer	No significant associations
Herpes simplex virus 1		Oropharyngeal carcinoma	Weak positive association
		Cervical cancer	
Human herpes virus -8 (Kaposi sarcoma)		Prostate cancer	No significant associations
		Non-Hodgkin Lymphoma	No significant associations
Varicella zoster virus		Glioma	Weak negative association
*T. vaginalis*		Prostate cancer	No significant associations
CMV	Anti-CMV	Gastrointestinal cancer	No significant associations
		Breast cancer	No significant associations
MCV	Anti-MCV	Merkel cell carcinoma	Weak positive association
		Bladder cancer	Weak positive association
BKV		Bladder cancer	Weak positive association
JCV		Bladder cancer	No significant association
		Colorectal cancer	No significant association
*Porphyromonas gingivalis*		Pancreatic cancer	Weak positive association
		Oropharyngeal cancer	Weak positive association
*Chlamydia pneumoniae*	IgA	Lung cancer	Weak positive association
Polyomavirus	Anti-polyomavirus	Non-Hodgkin lymphoma	No significant association
GBV	Anti-GBV	Non-Hodgkin lymphoma	No significant association
Propionibacterium Acnes		Prostate cancer	Intermediate positive association

VCA, viral capsid antigen; EA, early antigen; HBs, hepatitis B specific antigen; HBc, hepatitis B core antigen; \

### Epstein–Barr virus

Antibodies against four major EBV antigens (viral capsid antigen (anti-VCA) IgA, IgM and IgG, early antigen (anti-EA) IgG, EBV nuclear antigen (EBNA), and ZEBRA IgM) have been studied in association with the risk of various cancers. Several studies looking into the association between EBV immunoglobulins and nasopharyngeal carcinomas (NPC) have found a positive association with all EBV antigens. For instance, a cohort study found that anti-EBNA1 neutralizing antibodies may be a sensitive biomarker for risk of NPC [34]. This was supported by 2 other population-based studies [35, 36]. Moreover, a large cohort looking at anti-VCA IgA found a strong positive association with the risk of NPC [37]. These results were also supported by two other cohort studies [38, 39]. Of the five studies that examined the risk of gastric cancer (GC), two epidemiological studies reported an increased risk of GC in patients with positive anti-VCA IgG [40, 41]. However, no significant associations were found between anti-EA antibodies and risk of GC [42]. One case-control study with 321 cases of ovarian cancer reported a positive association with anti-EBV IgG, however, no association was found for anti-EBV IgA [43]. No significant associations have been reported between EBV antibodies and risk of lymphoma and breast cancer [44].

### Hepatitis B virus

Antibodies against hepatitis B virus antigens (hepatitis core antigen (anti-HBc) IgG, hepatitis B specific antigen (anti-HBs) IgG and IgM, and anti-hBe IgM) have long been suspected to be predictive factors for hepatocellular carcinoma (HCC). A consistent positive association was found between patients with HBsAg seropositive and risk of HCC [45]. In addition, three large cohort studies reported a stronger positive association in patients who were seropositive both anti-HBs and HBsAg compared with that seropositive for HBsAg [46]. Moreover, two population-based cohort studies found a positive association between positive anti-HBc antibodies and the risk of HCC [47, 48]. In contrast, a large cohort from a hepatitis B-endemic area found no significant association between patients with detected serum anti-HBs IgG and risk of HCC [49]. Furthermore, two case-control studies looking at anti-HCV, HBsAg, andi-HBc, and anti-HBs antibody positivity reported an increased risk of pancreatic cancer [50, 51]. No other associations were found between HBV antibodies and the risk of head and neck cancer and biliary tract cancer [52, 53].

### Hepatitis C virus

Several studies have investigated the relationship between hepatitis C virus antibodies and the risk of hepatocellular carcinoma. A case-control study found a strongly increased risk of HCC in patients with positive anti-HCV antibody seropositivity [54]. This positive association was supported by three other epidemiological studies [55–57]. Additionally, a cohort also analyzing HBV antibodies found a higher risk of HCC in individuals who were seropositive for antibodies to both HCV and HBV [55]. Moreover, three epidemiological studies looking into the association between HCV antibodies and lymphomas found no significant associations [58–60]. Lastly, a case–-control from Japan reported a positive association between anti-HCV antibodies and the risk of intrahepatic cholangiocarcinoma [61].

### Human papillomavirus

High risk (16 and 18) human papillomavirus antibodies have frequently been linked with cervical and other anogenital cancers (i.e. anus, vulvar, vaginal, and penile). Several epidemiological studies showed that serum antibodies to HPV 16 and 18 are associated with an increased risk of cervical cancer [62, 63]. Moreover, no consistent associations were found between other anogenital cancers and anti-HPV antibodies. Numerous studies investigating the relationship between HPV antibodies and head and neck cancers have found consistently positive associations between positive HPV 16 antibodies and the risk of head and neck cancers [64, 65]. No significant associations were found for other HPV types [64]. Furthermore, two large case-control studies from Sweden and Norway reported an increased risk of non-melanoma skin cancer in patients with detected antibodies for both HPV 16 and 18 [66, 67]. Lastly, no significant associations were found between HPV antibodies and the risk of prostate and lung cancer [68].

### Chlamydia trachomatis

Antibodies against chlamydia trachomatis have most commonly been associated with the risk of cancers of the reproductive system (i.e. ovarian, cervical, and prostate). A recent study using data from the EPIC cohort reported an increased risk of ovarian cancer in patients seropositive for antibodies recognizing chlamydia trachomatis [69]. A case-control found a positive association between high titers of antibodies against chlamydia trachomatis and cervical cancer; however, another large population-based case-control study found no significant associations [70]. Lastly, a case-control study with 38 incident cases of prostate cancer reported a protective effect in patients seropositive for chlamydia trachomatis antibodies [71].

### Helicobacter pylori

Most studies have focused on the association between *H. pylori* and the risk of gastric cancer. A recent cohort of 19 106 Japanese men reported an increased risk of GC for patients with undetectable anti-*H. pylori* IgG titers. However, the increase in risk was dependent on the severity of atrophic gastritis, resulting from persistent *H. pylori* infection [72]. This was supported by a cross-sectional study that found that serum IgG1 against *H. pylori* was significantly lower in subjects with GC (*n* = 62) [73]. On the other hand, a case-control study including 225 incident GC cases and 435 controls reported an increased risk of GC in individuals with elevated titers of IgA and IgG serum antibodies for *H. pylori* [74]. This positive association between immunoglobulin and risk of GC has been supported by the majority of epidemiological evidence to date [66–77]. Moreover, three epidemiological studies were identified looking at the association between antibodies against *H. pylori* and risk of colorectal cancer (CRC). However, no significant associations were found [78].

## Tumour and self-reactive antibodies

A total of 81 papers were identified assessing the risk of cancer in relation to tumour or self-reactive antibodies. Of the identified papers, 35 studies specifically addressed the risk of any cancer or specific types of cancers in the general population. A further 23 studies were directed towards specific ‘at risk’ populations, such as carriers of mutations in BReast CAncer gene (BRCA) or those with thyroid nodules, while another 23 addressed the risk of cancer in relation to various autoantibodies associated with autoimmune diseases within specific patient cohorts. An overview of the main findings from this section is given in Tables 4–6.

**Table 4:** Summary of results of associations between tumour and self-reactive antibodies and site-specific cancer risk, in the general population. The evidence of association is defined by the number of studies reporting on the association, the range of the hazard ratio/odds ratio/relative risks/standardized incidence ratio reported in each study, and the statistical significance.

Cancer type	Serum Antibodies	Main findings	Diagnostic potential
All/any	Anti-p53	Positive association	NA
	Anti-phospholipid	Possible inverse association	NA
Breast	Antibodies to six autoantigens: p53, c-myc, HER2, NY-ESO-1, BRCA2 and MUC1 (assessed individually)	Positive association for the presence of 1 or more of listed autoantibodies	Autoantibody panel likely to perform better than single marker – but not assessed
	Anti-thyroid peroxidase	Inverse association	NA
Colorectal	Anti-p53	Positive association	High specificity but low sensitivity
	Anti-Neu5Gc (antibodies to meat-derived antigens)	Positive association for total anti-Neu5Gc IgG; Single epitopes no association	NA
	IGFBP-2 IgG	Positive association	AUC = 0.92 (when combined with serum IGFBP-2 levels)
	Multiple TAA antibodies (8000 potential antigens)	Positive association: MAPKAPK3, PIM1, STK4, SRC, and FGFR4 Negative association: ACVR2B	Specificity and sensitivity high for anti-ACVR2B, anti-MAPKAPK3, anti-PIM1 combined
	Anti-ASXL2*	Positive association	AUC = 0.67
Oesophageal	Anti-ASXL2*	Positive association	AUC = 0.76
	Anti-p53	Positive association	NA
Gastric	Panel; p62, c-Myc, NPM1, 14-3-3ξ, MDM2 and p16	Positive associations	Selected six panel for testing
Glioma	Anti-IGFBP-2	Positive association for astrocytoma	AUC = 0.80 (when combined with serum IGFBP-2 levels)
Hepatocellular carcer	Multiple TAA antibodies	Positive associations for autoantibodies to calreticulin, cytokeratin 8, nucleoside diphosphate kinase A, F1-ATP synthase	NA
	Multiple antibodies	Positive association for antibodies to 21 TAAs; (best performers: IMP-1, KOC, p53 and c-myc, Sui1 and RalA, Calreticulin, and HCC1)	Moderate sensitivity high specificity
	12 antibody panel	Positive association for autoantibodies to HCC1, P16, P53, P90, and Survivin	NA
Lung cancer/NSCLC	Panel: p62, BIRC, Livin-1, p53, PRDX, NY-ESO-1 and Ubiquitin	Positive association	AUC = 0.81
	Panel: p53, c-myc, HER2, NY-ESO-1, CAGE, MUC1 ans GBU4-5	Positive association	High sensitivity for squamous cell lung cancer, moderate sensitivity for all lung cancers
	Panel: GAGE7, CAGE, MAGEA1, SOX2, GBU4-5, PGP9.5, and p53	Positive association	Moderate sensitivity and specificity
	Multiple antibodies: (212 selected from immunogenic tumour expressed proteins)	Positive association for the 5 most immunogenic combined	High sensitivity and specificity
	Multiple autoantibodies: p62, p16, Koc, p53, Cyclin B1, Cyclin E, Survivin, HCC1, and RalA	Strongest serological response: Survivin, Cyclin B1, HCC1, and p53	Low to moderate potential as individual autoantibodies
	Anti-BARD1	Positive association	High sensitivity and specificity
	Brain protein autoantibodies	Possible positive association	NA
	CD25 and FoxP3 IgGs	Positive association for CD25a; weaker for FoxP3	NA
Mesothelioma	Panel including PDIA6, MEG3, SDCCAG3, IGHG3, IGHG1	Positive association	High specificity and sensitivity
	Anti-p53	No association	NA
Lymphoma/NHL	Anti-cyclic citrullinated peptide (CCP)	No association	NA
	Autoimmune diseases No individual autoantibodies specified	Positive association	NA
Ovarian cancer	Panel: anti- MDM2, PLAT, NPM1, 14-3-3 Zeta, p53, and RalA	Positive association	High specificity and sensitivity
	Anti-MUC1, anti-CA125	Positive association	NA
	Anti-MUC1	Indirect evidence for inverse association	NA
	Anti-p53 and anti-SBP-1**	Positive association for serous ovarian cancer	High specificity and sensitivity for CA125, anti-TP53, and anti-SBP1 combined (AUC 0.96)
	Anti-HE4	Positive association	NA
Pancreatic	Anti-Ezrin	No association	NA
Bladder	Anti-UPII	Positive association	

Regarding the target antigens for the autoantibodies evaluated in the general population (Table 4), studies generally showed positive associations for single-antigen targets such as p53, New5Gc, IGFBP-2, BARD1, CD25, FoxP3, MUC1, CA125, SBP1, and HE4, albeit with relatively low sensitivity [79–87]. The remaining 13 studies investigated the general population to assess cancer risk in relation to serum antibodies against multiple antigens, either to identify the most immunogenic of these or to develop and evaluate a panel of biomarkers for diagnostic or screening purposes. These studies indicate strong positive associations for multiple antibodies, with most indicating relatively high specificity and sensitivity for specific antibody panels against tumour specific and self-antigens [88]. For example, Zhang et al. reported a panel of nine candidate autoantibody markers that achieved 94.3% sensitivity and 90.4% specificity for detecting mesothelioma [89]. Similarly, a panel comprising antibodies to the autoantigens, p53, c-myc, human epidermal growth factor receptor 2 (HER2), New York Esophageal Squamous Cell Carcinoma-1 (NY-ESO-1), cancer/testis antigen gene (CAGE), Mucin 1 (MUC1), and GBU4-5, achieved high sensitivity and specificity for the detection of squamous cell lung cancers [90].

Studies performed within specific target populations included populations at high cancer risk (e.g. BRCA mutation carriers, populations identified with high risk of oesophageal cancer for screening), cohorts with specific organ disease populations (e.g. lung and thyroid disease, and populations with autoimmune or paraneoplastic syndromes, including autoimmune myopathies, scleroderma, Sjorgens syndrome, autoimmune thyroiditis, autoimmune vasculitis, and autoimmune phemphoid) (Tables 5 and 6). The most consistent positive associations were seen for the association between overall cancer risk in scleroderma patients with anti-RNA polymerase-3 (RNAP-3) antibodies and for thyroid cancer among people with autoimmune thyroiditis who had high anti-thyroglobulin (Tg) serum antibody titres [91, 92]. Among people undergoing thyroidectomy for any reason, thyroid cancer risk was also associated with the presence of anti-Tg antibodies, though the evidence was mixed regarding anti-thyroid peroxidase (TPO) seropositive status [93]. Antibody panels applied in high-risk populations again appeared to have relatively high sensitivity and specificity in relation to identifying patients with cancer or individuals with premalignant disease [8].

**Table 5:** : Summary of results of associations between tumour and self-reactive antibodies and site-specific cancer risk, in specific populations. The evidence of association is defined by the number of studies reporting on the association, the range of the hazard ratio/odds ratio/relative risks/standardized incidence ratio reported in each study, and the statistical significance.

Target Population	Cancer risk	Serum antibody	Main findings	Diagnostic potential
High risk (BRCA) carriers	Breast cancer	MUC1 IgG	No association	NA
High risk oesophageal cancer (screening) population	Oesophageal cancer	Panel of eight autoantibodies: p53, IMP1, P16, cyclin B1, P62, c-myc, Survivn and Koc NY-ESO-1 STIP1	Positive association	High specificity and moderate sensitivity
Lung disease	Premalignant lung lesions Atypical adenomatous hyperplasia/ squamous cell dysplasia	Panel of nine autoantibodies	Positive association	Moderate specificity and moderate sensitivity for premalignancy
Endometrial cancer patients	Endometrial cancer	Anti-p53	Positive association for serous histology	NA
Ovarian cancer patients	Ovarian cancer	NY-ESO-1	48% seropositive	NA
Thyroid disease: (patients having nodule FNAB or thyroidectomy)	Thyroid cancer (papillary carcinoma)	Anti-Tg	Positive association No association	High specificity and low sensitivity (ref 80)
		Anti-TPO	Positive association No association	NA
		Autoimmune thyroiditis (either Tg or TPO Ab +ve)	No association	NA

**Table 6:** Summary of results of associations between autoimmune diseases and site-specific cancer risk. The evidence of association is defined by the number of studies reporting on the association, the range of the hazard ratio/odds ratio/relative risks/standardized incidence ratio reported in each study, and the statistical significance.

Autoimmune diseases	Cancer risk	Serum antibody	Main findings
Autoimmune diseases (any)	Any cancer, specific cancers	Anti-Ro/SSA	Positive association for risk of any cancer, melanoma, lymphoma, breast cancer
Celiac disease (Undiagnosed)	Any cancer	IgA-TTG; IgG TTG levels	Positive association
Autoimmune encephalitis	Any cancer	Anti-MNDAW	Cancer prevalence 6%
Autoimmune myopathies	Any cancer (concomitant)	Positive Anti-SRP; positive anti-HMGCR	Positive association for anti- HMGCR for necrotizing myopathies
	Any cancer (concomitant)	Myositis specific antibodies (MSAs): anti-TIF1-γ, anti-NXP2, anti-SAE1,	Positive association for inflammatory myositis with any single MSA +ve; positive association for MSAs -ve
	Any cancer (concomitant)	Anti-MJ/NXP-2, anti-MDA5, and Anti-TIF1γ/α	Positive association for anti-TIF1-γ in dermatomyositis
	Lung Ca	Anti-TIF1; anti-NXP2; anti-RNAP3	No association
Scleroderma	Any cancer (concomitant)	Anti-centromere	Negative association [3], No association [1]
		Anti-RNAP-3	Positive association
		Anti-TOPO	Weaker positive association
		Anti-RNAP-1 (large subunit)	Inverse association
		Anti–RNPC-3	Positive association
	Lung cancer	Anti-centromere Anti-TOPO	No association
	Breast cancer (concomitant)	Anti-RNAP3	Positive association
Sjogren’s syndrome	Hodgkin disease	Anti-centromere	No association
	Lymphoma	Ro/SSA and La/SSB	No Association
	MALT NHL	Ro/SSA and La/SSB	No Association
	Myeloma	Ro/SSA and La/SSB	Positive (suggestive)
Autoimmune Thyroiditis	Thyroid ca	Anti-Tg	Positive association No association
		Anti-TPO	Positive association No Association
		TPOAb and TgAb combined	Strong positive association
Autoimmune Vasculitis	Any cancer	ANCA- vasculitis	No association/inconclusive
Autoimmune oral phemphoid patients	Any cancer	Anti-alpha6-integrin	Inverse association

## Discussion

There is a growing body of evidence linking antibodies to cancer risk, especially for specific immunoglobulin isotypes and for both TAA-reactive and self-reactive antibodies. B cells can suppress tumour growth by producing antibodies able to facilitate CDC and antigen presentation or engage effector cells to mediate ADCC and ADCP; however, have shown the ability to also support tumour growth by expressing antibodies with poor ability to mediate the above anti-tumour responses, such as isotypes like IgG2 and IgG4 [6]. Antibodies have great potential as biomarkers for cancer since procedures to test for their presence are minimally invasive and antibodies are fairly stable and easy to measure *ex vivo*. Research identifying antibodies that might be appropriate biomarkers for early detection of cancer, based on our systematic review, still appears to be an emerging field. Most of the studies reviewed were observational, and largely attempted to identify potential markers in association with cancer. Few of the studies test the predictive potential of antibodies to detect an overall cancer risk or risk for site-specific cancers, specifically for one or more autoantibodies (autoantibody panels). Larger studies are required to validate these antibodies as cancer biomarkers and to apply them in clinical practice. The current systematic review presents a broad landscape of potential biomarkers for early diagnosis of cancer and our findings highlight the importance of this newly emerging research topic in cancer biomarker discovery.

A consistent association between serum levels of certain immunoglobulin isotypes and risk for certain cancers was found. However, studies have been conducted in several methods and in different settings and have reported diverse results. It is therefore difficult to point to antibody class-specific associations with cancer risk. While no association was found between general IgG and IgM levels, several studies we found report associations between altered serum levels of IgE and cancer risk [13, 15, 19, 21, 22]. Some studies report that high titres of allergen-specific IgE are associated with an increased risk of prostate cancer and squamous cell carcinoma, and conversely these show a negative association with glioma incidence [29, 30]. Low IgE titres have also been associated with a high risk of overall cancer and an increased risk of haematological malignancies [25]. High IgA titres have been found associated with the risk of a range of solid tumours, but have a strong negative association with risk of gastrointestinal cancer and lymphoma diagnosis, while other studies show no significant associations with overall, pancreatic, melanoma, and bladder cancer risk, in relation to IgA levels [19, 20].

Different types of cancers have different aetiology. This might explain the controversial results on different effects of IgE and IgA antibody titres on different types of cancers [19, 24]. IgE can exert anti-tumour functions, but can be also associated with systemic chronic inflammation, which can instead promote tumourigenesis [21]. This could explain the association between general low serum levels of IgE and increased cancer risk for cancers located far from the site of inflammation. On the other hand, high IgE levels as part of the local chronic inflammatory milieu could predispose to cancers developing at that site of inflammation; for example lung cancer in patients with asthma and non-melanoma skin cancers in patients with atopic dermatitis [28]. IgA may have a protective role in certain tissues such as mucosal areas and the gastrointestinal tract, which would explain the strong negative correlation between high IgA levels and positive associations with the development of gastrointestinal cancer [14, 19]. However, IgA can be associated with specific B cell subsets and their regulatory functions, such as the production of immunosuppressive cytokines like IL-10, which can support a pro-tumour microenvironment [19]. Together, these studies may suggest the importance of considering the inflammatory environment in disparate anatomical sites and its relation to the nature of the humoral response and how this might relate to carcinogenesis.

With the exception of antibodies against EBV which have shown a positive association with NPCs, and possibly with gastric and ovarian cancers, most studies focused on investigating the association between the presence of anti-virus or anti-bacteria antibodies and cancer risk, report a positive association with specific cancers known to be associated to that specific virus/bacterial infection site (for instance, HBV and HCV with hepatocellular carcinoma, HPV with anogenital cancers), but no association with other cancers [34, 39, 42]. Testing the presence of specific anti-viral or anti-bacterial antibodies is often the only method to assess if there is or there has been a specific infection, and it is therefore difficult to distinguish whether it is the presence of the antibodies that is associated with cancer risk and not the virus/bacterial infection itself [72]. In addition, only one study relating to infectious agents described the presence of neutralizing antibodies (i.e. EBV) [34]. The presence of neutralizing antibodies implies that the humoral response generated is already protective against a pathogen, compared to an antibody response that recognizes the epitope but which still allows the pathogen to infect, survive, and replicate. Therefore, further studies looking into the association between neutralizing antibodies against various infectious agents in association to cancer risk are required.

The risk of cancer appeared to be increased among people who present with various autoimmune diseases (i.e. in most cases autoimmune diseases involve the body producing antibodies toward ‘self’ (autoantigens) which can lead to local or systemic inflammation and specific or systemic organ damage) [90, 93]. However, despite the increased risk of cancer, those with autoimmune diseases often have a better prognosis, leading to the hypotheses that such immune responses may be protective against the development of autoantigen-expressing cancer cells at an early stage of carcinogenesis, and therefore preventing cancer from developing and progressing [94]. Evidence of a better prognosis has been found for the development of vitiligo, denoting an immune attack on melanocytes, in patients with melanoma, and of thyroiditis in patients with thyroid cancer [10].

Several studies indicate a high risk of cancer diagnosis within 3 years of diagnosis of specific autoimmune diseases, specifically scleroderma and autoimmune myopathies, suggesting that some autoimmune pathologies may actually represent a ‘paraneoplastic process’ [91]. One hypothesis is that the loss of tolerance to ‘self’ may be due to cross-reaction with tumour autoantigens, leading to the immune system targeting non-cancerous tissue in the process of mounting an immune response to the tumour. Hence the development of autoimmune disease could be an early marker for cancer development prior to becoming clinically detectable [95]. This has prompted calls for cancer screening in patients recently diagnosed with specific autoimmune diseases. However, we did not find examples of any specific autoantibodies being trialled as biomarkers to evaluate cancer risk among patient populations with autoimmune diseases. This may be due to their rather low sensitivity for use as a stand-alone screening tool.

Over the past few decades, it is evident that a substantial body of work has been undertaken to identify the most immunogenic TAAs and their associated tumour-reactive antibodies and autoantibodies, with the end goal being the development of assays to measure the presence of such antibodies (alone or in combination with other markers) for use in cancer screening and diagnostics [94]. Many of these studies have focussed on cancers where screening tests are currently lacking and, or in tumour types such as e.g. in lung cancers, hepatocellular carcinomas, and ovarian cancers in which earlier detection and prompt clinical intervention could provide substantial survival benefits [82, 83, 89, 90].

Some autoantibodies were frequently observed in association with multiple cancer types for example anti-p53 antibodies are detected in breast, colorectal oesophageal, lung, and ovarian cancers [88, 96]. While the detection of anti-p53 antibodies alone is not a very sensitive marker for cancer, a combination with other antibody biomarkers and clinical characteristics could provide additional value for risk stratification [85]. Anti-MUC1 antibodies mark another autoantibody found to be associated with several cancer types and appear to correlate with a more favourable prognosis [82]. MUC1 (also known as CA 15.3) is a transmembrane mucin, a glycoprotein with O-glycosylated tandem repeats, overexpressed in cancer, in particular in breast cancer. MUC1 has been also found aberrantly glycosylated in cancer compared to normal tissue, and some reported anti-MUC1 antibodies are actually against these aberrantly glycosylated variants [97]. Anti-MUC1 antibodies MUC1 have shown a positive association with breast cancer when in combination with 1 or more of the listed autoantibodies p53, c-myc, HER2, NY-ESO-1, BRCA2 [87]. Anti-MUC1 IgG1 antibodies have also shown a positive association with ovarian cancer [81]. Another report on lung cancer shows a positive association with high sensitivity for squamous cell lung cancer and moderate sensitivity for all lung cancers, for an autoantibody panel which includes MUC1 (p53, c-myc, HER2, NY-ESO-1, CAGE, and MUC1, GBU4-5) [90]. Of interest are also antibodies targeting cyclin B1, a protein involved in the transition from G2 to M phase of the cell cycle. Anti-cyclin B1 antibodies have been found to increase in lung cancer [98], with low to moderate prediction potential as individual autoantibody, and oesophageal carcinoma, with high specificity and moderate sensitivity in predicting cancer risk when in a panel with other 7 autoantibodies (p53, IMP1, P16, P62, c-myc, Survivn, and Koc) [99]. However, in patients with breast cancer they have been found to decrease compared to healthy volunteers and they were actually considered to have a protective role in breast cancer development [100]. Given that many identified autoantibodies to one antigen have low sensitivity and/or specificity as biomarkers for individual cancer types, the development of panels of multiple autoantibodies may provide better sensitivity for cancer or specific cancer types.

This systematic review provides a comprehensive qualitative summary of the published epidemiological evidence of the associations between antibodies and the risk of overall and site-specific cancers. A large number of the cohort, case-control studies were included. However, given the broad subject and large amount of antibody types, in this study, we presented an overview of the most researched antibodies in relation to cancer risk, and it is possible that certain studies might have been missed in our literature review. Our systematic review thus presents a broad landscape of different antibodies with the potential of being identified and in the future validated as markers of early diagnosis of cancer. Larger observational studies and clinical trials are necessary to establish the potential prediction capability of such biomarkers or their combinations.

## Conclusion

There is consistent evidence associating antibodies to cancer risk, especially for specific immunoglobulin isotypes and for both tumour-associated and self-reactive antibodies. However, research in this field is still in the early stages of development. No clinical trials assessing the utility of antibodies as biomarkers for screening or diagnostic assessment were identified, and most of the studies thus far have reported exploratory findings from observational studies, some involving modeling with validation cohorts with reports of specificity and sensitivity but which still require validation with larger cohorts. Therefore, larger studies and clinical trials are necessary to assess the efficacy of specific antibodies as markers for early cancer diagnosis.

## Supplementary Material

uxac030_suppl_Supplementary_Table_S1

## Data Availability

The data presented in this study are available on request from the corresponding author.

## Abbreviations

ADCC: antibody-dependent cell cytotoxicity
ADCP: antibody-dependent cell phagocytostis
ANCA: antineutrophil cytoplasmic antibody
Anti-EA: early antigen
Anti-HBc: hepatitis core antigen
Anti-HBs: hepatitis B specific antigen
Anti-UPII: uroplakin II antibody
Anti-VCA: viral capsid antigen
ASXL2: additional sex combs like 2
BARD1: BRCA1-associated RING domain protein 1
BKV: BK virus
BIRC: baculoviral inhibitors of apoptosis repeat containing
BRCA: breast cancer gene
CagA: cytotoxic associated gene A
CAGE: cancer/testis antigen gene
CA125: cancer antigen 125
CDC: complement-dependent cytotoxicity
CMV: cytomegalovirus
CRC: colorectal cancer
C1q: complement component 1q
EBNA: Epstein-Barr nuclear antigen
EBV: Epstein-Barr virus
EPIC: European Prospective Investigation into Cancer and Nutrition
FcRs: FC receptors
FNAB: fine needle aspiration biopsy
FOXP3: forkhead box P3
GAGE7: G Antigen 7
GBV: Great Britain virus
GC: gastric cancer
HBV: hepatitis B virus
HCC: hepatocellular carcinoma
HCV: hepatitis C virus
HE4: human epididymis protein 4
HER2: human epidermal growth factor receptor 2
HP: helicobacter pylori
HPV: human papillomavirus
Ig: immunoglobulin
IgA: immunoglobulin A
IgE: immunoglobulin E
IGFBP: insulin-like growth factor-binding protein
IgG: immunoglobulin G
IgG4-RD: immunoglobulin G4 related disease
IgM: immunoglobulin M
JCV: John Cunningham virus
La/SSB: Sjögren’s-syndrome-related antigen B
MAGEA1: melanoma-associated antigen 1
MCV: molluscum contagiosum virus
MDM2: mouse double minute 2 homolog
MEG3: maternally expressed gene 3
mTSA: mutated tumour specific antigen
MUC1: mucin 1
Neu5Gc: N-glycolylneuraminic acid
nmTAA: non-mutational tumour-associated antigen
NPC: nasopharyngeal carcinoma
NPM1: nucleophosmin
NXP2: nuclear matrix protein
NY-ESO-1: New York esophageal squamous cell carcinoma-1
PDIA6: protein disulfide isomerase family A: member 6
PGP9.5: protein gene product 9.5
PLAT: tissue plasminogen activator
PRDX: peroxiredoxins
PRISMA: preferred reporting items for systematic reviews and meta-analyses
PSA: prostate-specific antigen
RalA: Ras-related protein Ral-A
RD: related disease
RNAP: ribonucleic acid polymerase
RNP-3: U11/U12 small nuclear ribonucleoprotein
Ro/SSA: Sjögren’s-syndrome-related antigen A
RPA: replication protein A
SAE: small ubiquitin-like modifier activating enzyme
SBP1: selenium-binding protein 1
SCCA: squamous cell carcinoma antigen
SDCCAG3: serologically defined colon cancer antigen-3
SR: self-reported
SRP: anti-signal recognition particle
TAA: tumour-associated antigen
Tg: anti-thyroglobulin
TIF1: transcriptional intermediary factor
TOPO: topoisomerase
TPO: anti-thyroid peroxidase
TTG: tissue transglutaminase
VacA: vacuolating cytotoxin
