# Supplementary material for: Antibodies as biomarkers for cancer risk: a systematic review
Source: Clin Exp Immunol. 2022 Apr 4;209(1):46–63. doi: 10.1093/cei/uxac030 (PMC9307228; doi:10.1093/cei/uxac030)
Supplement: uxac030_suppl_Supplementary_Table_S1 [file uxac030_suppl_supplementary_table_s1.docx]

Supplementary Table 1. Studies included in results section (tables 2-6).

| First  Author | Year | Title | Journal |
| --- | --- | --- | --- |
| Immunoglobulin isotypes | | | |
| Mellemkjaer L | 2002 | Cancer risk among patients with IgA deficiency or common variable immunodeficiency and their relatives: a combined Danish and Swedish study | Clin Exp Immunol |
| Kyle RA | 2003 | Long-term follow-up of IgM monoclonal gammopathy of undetermined significance | Semin Oncol |
| Lindelöf B | 2005 | Allergy and cancer | Allergy |
| Wang H | 2006 | Atopic diseases, immunoglobulin E and risk of cancer of the prostate, breast, lung and colorectum | Int J Cancer |
| Pontisso P | 2006 | Progressive increase of SCCA-IgM immune complexes in cirrhotic patients is associated with development of hepatocellular carcinoma | Int J Cancer |
| Petridou ET | 2007 | Breast cancer risk in relation to most prevalent IgE specific antibodies: a case control study in Greece | Anticancer Res |
| Melbye M | 2007 | Atopy and risk of non-Hodgkin lymphoma | J Natl Cancer Inst |
| Chang JS | 2009 | Maternal immunoglobulin E and childhood leukemia | Cancer Epidemiol Biomarkers Prev |
| Van Hemelrijck M | 2010 | Immunoglobulin E and cancer: a meta-analysis and a large Swedish cohort study | Cancer Causes Control |
| Vajdic CM | 2010 | Are antibody deficiency disorders associated with a narrower range of cancers than other forms of immunodeficiency? | Blood |
| Wiemels JL | 2011 | Risk of squamous cell carcinoma of the skin in relation to IgE: a nested case-control study | Cancer Epidemiol Biomarkers Prev |
| Chapman CJ | 2011 | Immunobiomarkers in small cell lung cancer: potential early cancer signals | Clin Cancer Res |
| Schlehofer B | 2011 | Primary brain tumours and specific serum immunoglobulin E: a case-control study nested in the European Prospective Investigation into Cancer and Nutrition cohort | Allergy |
| Freeman JA | 2013 | Immunoglobulin G subclass deficiency and infection risk in 150 patients with chronic lymphocytic leukemia | Leuk Lymphoma |
| Svensson T | 2013 | Clinical significance of serum immunoglobulin G subclass deficiency in patients with chronic lymphocytic leukemia | Scand J Infect Dis |
| Amirian ES | 2013 | Antihistamine use and immunoglobulin E levels in glioma risk and prognosis | Cancer Epidemiol |
| Lariou MS | 2013 | Allergy and risk of acute lymphoblastic leukemia among children: a nationwide case control study in Greece | Cancer Epidemiol |
| Smorodin E | 2013 | The relation of the level of serum anti-TF, -Tn and -alpha-Gal IgG to survival in gastrointestinal cancer patients | Int J Med Sci |
| Ishida M | 2013 | Occurrence of anaplastic large cell lymphoma following IgG4-related autoimmune pancreatitis and cholecystitis and diffuse large B-cell lymphoma | Int J Clin Exp Pathol |
| Skaaby T | 2014 | Atopy and development of cancer: a population-based prospective study | J Allergy Clin Immunol Pract |
| Olson SH | 2014 | Serum immunoglobulin e and risk of pancreatic cancer in the prostate, lung, colorectal, and ovarian cancer screening trial | Cancer Epidemiol Biomarkers Prev |
| Taşli F | 2014 | The role of IgG4 (+) plasma cells in the association of Hashimoto's thyroiditis with papillary carcinoma | APMIS |
| Nieters A | 2014 | Prediagnostic immunoglobulin E levels and risk of chronic lymphocytic leukemia, other lymphomas and multiple myeloma-results of the European Prospective Investigation into Cancer and Nutrition | Carcinogenesis |
| de Costa D | 2014 | Peptides from the variable region of specific antibodies are shared among lung cancer patients | PLoS One |
| Li N | 2014 | IL17A gene polymorphisms, serum IL-17A and IgE levels, and hepatocellular carcinoma risk in patients with chronic hepatitis B virus infection | Mol Carcinog |
| An G | 2014 | Polyclonal serum IgM level identifies a subgroup of multiple myeloma patients with low-risk clinicobiological features and superior survival | Leuk Res |
| Ludvigsson JF | 2015 | IgA deficiency and risk of cancer: a population-based matched cohort study | J Clin Immunol |
| Hollander P | 2015 | Autoimmune and Atopic Disorders and Risk of Classical Hodgkin Lymphoma | Am J Epidemiol |
| Bojková M | 2015 | Immunoglobulin G4, autoimmune pancreatitis and pancreatic cancer | Dig Dis |
| Helby J | 2015 | IgE and risk of cancer in 37 747 individuals from the general population | Ann Oncol |
| Liao HC | 2016 | Allergy symptoms, serum total immunoglobulin E, and risk of head and neck cancer | Cancer Causes Control |
| Biasiolo A | 2016 | Squamous cell carcinoma antigen-IgM is associated with hepatocellular carcinoma in patients with cirrhosis: A prospective study | Dig Liver Dis |
| Ahn SS | 2017 | Malignancies in Korean patients with immunoglobulin G4-related disease | Int J Rheum Dis |
| Van Hemelrijck M | 2017 | Atopy and prostate cancer: Is there a link between circulating levels of IgE and PSA in humans? | Cancer Immunol Immunother |
| Ferastraoaru D | 2018 | IgE deficiency and prior diagnosis of malignancy: Results of the 2005-2006 National Health and Nutrition Examination Survey | Ann Allergy Asthma Immunol |
| Chen X | 2018 | A genome-wide association study of IgM antibody against phosphorylcholine: shared genetics and phenotypic relationship to chronic lymphocytic leukemia | Hum Mol Genet |
| Kantor ED | 2019 | Allergies and Asthma in Relation to Cancer Risk | Cancer Epidemiol Biomarkers Prev |
| Li N | 2019 | Value of immune factors for monitoring risk of lung cancer in patients with interstitial lung disease | J Int Med Res |
| Glenn MJ | 2019 | Elevated IgM and abnormal free light chain ratio are increased in relatives from high-risk chronic lymphocytic leukemia pedigrees | Blood Cancer J |
| Aussy A | 2019 | The IgG2 Isotype of Anti-Transcription Intermediary Factor 1γ Autoantibodies Is a Biomarker of Cancer and Mortality in Adult Dermatomyositis | Arthritis Rheumatol |
| Infection-related antibodies | | | |
| Jackson LA | 2000 | Association of Chlamydia pneumoniae immunoglobulin A seropositivity and risk of lung cancer | Cancer Epidemiol Biomarkers Prev |
| Iaquinto G | 2000 | Antibody response to Helicobacter pylori CagA and heat-shock proteins in determining the risk of gastric cancer development | Dig Liver Dis |
| Carter JJ | 2001 | Human papillomavirus 16 and 18 L1 serology compared across anogenital cancer sites | Cancer Res |
| Starr JR | 2001 | Serologic evidence of herpes simplex virus 1 infection and oropharyngeal cancer risk | Cancer Res |
| Tulinius H | 2001 | Helicobacter pylori antibodies and gastric cancer in Iceland - The decline in IgG antibody level is a risk factor | APMIS |
| Fujioka N | 2001 | Serological Immunoglobulin G antibody titers to Helicobacter pylori in Japanese Brazilian and Non-Japanese Brazilian gastric cancer patients and controls in São Paulo | Jpn J Cancer Res |
| Mork J | 2001 | Human papillomavirus infection as a risk factor for squamous-cell carcinoma of the head and neck | N Engl J Med |
| Omer RE | 2001 | The role of hepatitis B and hepatitis C viral infections in the incidence of hepatocellular carcinoma in Sudan | Trans R Soc Trop Med Hyg |
| Shmuely H | 2001 | Relationship between Helicobacter pylori CagA status and colorectal cancer | Am J Gastroenterol |
| Limburg P | 2001 | Helicobacter pylori seropositivity and subsite-specific gastric cancer risks in Linxian, China | J Natl Cancer Inst |
| Hisada M | 2001 | Prospective study of antibody to human papilloma virus type 16 and risk of cervical, endometrial, and ovarian cancers (United States) | Cancer Causes Control |
| Groves FD | 2002 | Serum antibodies to Helicobacter pylori and the CagA antigen do not explain differences in the prevalence of precancerous gastric lesions in two Chinese populations with contrasting gastric cancer rates | Cancer Epidemiol Biomarkers Prev |
| Limburg PJ | 2002 | Helicobacter pylori seropositivity and colorectal cancer risk: a prospective study of male smokers | Cancer Epidemiol Biomarkers Prev |
| Nomura AM | 2002 | Helicobacter pylori CagA seropositivity and gastric carcinoma risk in a Japanese American population | J Infect Dis |
| Cheng WM | 2002 | Assessing the risk of nasopharyngeal carcinoma on the basis of EBV antibody spectrum | Int J Cancer |
| Yang HI | 2002 | Hepatitis B e antigen and the risk of hepatocellular carcinoma | N Engl J Med |
| Smith JS | 2002 | Herpes simplex virus-2 as a human papillomavirus cofactor in the etiology of invasive cervical cancer | J Natl Cancer Inst |
| Yamaji Y | 2002 | Weak response of helicobacter pylori antibody is high risk for gastric cancer: a cross-sectional study of 10,234 endoscoped Japanese | Scand J Gastroenterol |
| Chang YW | 2002 | Role of Helicobacter pylori infection among offspring or siblings of gastric cancer patients | Int J Cancer |
| Dahlstrom, KR | 2003 | Human papillomavirus type 16 infection and squamous cell carcinoma of the head and neck in never-smokers: a matched pair analysis | Clin Cancer Res |
| Adami HO | 2003 | Prostate cancer risk and serologic evidence of human papilloma virus infection: a population-based case-control study | Cancer Res |
| Littman AJ | 2003 | Association between late age at infectious mononucleosis, Epstein-Barr virus antibodies, and ovarian cancer risk | Scand J Infect Dis |
| Lehtinen M | 2003 | Evaluation of antibody response to human papillomavirus early proteins in women in whom cervical cancer developed 1 to 20 years later | Am J Obstet Gynecol |
| Di Bisceglie AM | 2003 | Hepatitis C-related hepatocellular carcinoma in the United States: influence of ethnic status | Am J Gastroenterol |
| Imazeki F | 2003 | Significance of prior hepatitis B virus infection in the development of hepatocellular carcinoma in patients with chronic hepatitis C | Dig Dis Sci |
| Sun CA | 2003 | Incidence and cofactors of hepatitis C virus-related hepatocellular carcinoma: a prospective study of 12,008 men in Taiwan | Am J Epidemiol |
| de Sanjose S | 2004 | Helicobacter pylori and malignant lymphoma in Spain | Cancer Res |
| Pickard A | 2004 | Epstein-Barr virus seroreactivity among unaffected individuals within high-risk nasopharyngeal carcinoma families in Taiwan | Int J Cancer |
| de Sanjose S | 2004 | Role of hepatitis C virus infection in malignant lymphoma in Spain | Int J Cancer |
| Richardson AK | 2004 | Cytomegalovirus, Epstein-Barr virus and risk of breast cancer before age 40 years: a case-control study | Br J Cancer |
| Yamamoto S | 2004 | Hepatitis C virus infection as a likely etiology of intrahepatic cholangiocarcinoma | Cancer Sci |
| Newton R | 2004 | Human papillomaviruses and cancer in Uganda | Eur J Cancer Prev |
| Hann HW | 2004 | Preneoplastic markers of hepatitis B virus-associated hepatocellular carcinoma | Cancer Res |
| Janulaityte-Günther D | 2005 | Helicobacter pylori antibodies and gastric cancer: a gender-related difference | FEMS Immunol Med Microbiol |
| Kosunen TU | 2005 | Association of Helicobacter pylori IgA antibodies with the risk of peptic ulcer disease and gastric cancer | World J Gastroenterol |
| Korodi Z | 2005 | No serological evidence of association between prostate cancer and infection with herpes simplex virus type 2 or human herpesvirus type 8: a nested case-control study | J Infect Dis |
| Korodi Z | 2005 | Human papillomavirus 16, 18, and 33 infections and risk of prostate cancer: a Nordic nested case-control study | Cancer Epidemiol Biomarkers Prev |
| Anttila T | 2005 | Chlamydial antibodies and risk of prostate cancer | Cancer Epidemiol Biomarkers Prev |
| Watabe H | 2005 | Predicting the development of gastric cancer from combining Helicobacter pylori antibodies and serum pepsinogen status: a prospective endoscopic cohort study | Gut |
| Knekt P | 2006 | Helicobacter pylori IgA and IgG antibodies, serum pepsinogen I and the risk of gastric cancer: changes in the risk with extended follow-up period | Int J Cancer |
| Sutcliffe S | 2006 | Plasma antibodies against Trichomonas vaginalis and subsequent risk of prostate cancer | Cancer Epidemiol Biomarkers Prev |
| Franceschi S | 2006 | Hepatitis C virus and non-Hodgkin's lymphoma: Findings from the Swiss HIV Cohort Study | Br J Cancer |
| Con SA | 2006 | Helicobacter pylori CagA status associated with gastric cancer incidence rate variability in Costa Rican regions | J Gastroenterol |
| Sasazuki S | 2006 | Effect of Helicobacter pylori infection combined with CagA and pepsinogen status on gastric cancer development among Japanese men and women: a nested case-control study | Cancer Epidemiol Biomarkers Prev |
| Vorobjova T | 2006 | Response of IgG1 and IgG2 subclasses to Helicobacter pylori in subjects with chronic inflammation of the gastric mucosa, atrophy and gastric cancer in a country with high Helicobacter pylori infection prevalence | APMIS |
| Kamangar F | 2006 | Opposing risks of gastric cardia and noncardia gastric adenocarcinomas associated with Helicobacter pylori seropositivity | J Natl Cancer Inst |
| Tanaka K | 2006 | Antibody to hepatitis B core antigen is associated with the development of hepatocellular carcinoma in hepatitis C virus-infected persons: a 12-year prospective study | Int J Mol Med |
| Janulaityte-Günther D | 2007 | Combined serum IgG response to Helicobacter pylori VacA and CagA predicts gastric cancer | FEMS Immunol Med Microbiol |
| Friborg J | 2007 | Epstein-Barr virus immune response in high-risk nasopharyngeal carcinoma families in Greenland | J Med Virol |
| de Sanjosé S | 2007 | Epstein-Barr virus infection and risk of lymphoma: immunoblot analysis of antibody responses against EBV-related proteins in a large series of lymphoma subjects and matched controls | Int J Cancer |
| Suzuki G | 2007 | Low-positive antibody titer against Helicobacter pylori cytotoxin-associated gene A (CagA) may predict future gastric cancer better than simple seropositivity against H. pylori CagA or against H. pylori | Cancer Epidemiol Biomarkers Prev |
| Palli D | 2007 | CagA+ Helicobacter pylori infection and gastric cancer risk in the EPIC-EURGAST study | Int J Cancer |
| Madeleine MM | 2007 | Risk of cervical cancer associated with Chlamydia trachomatis antibodies by histology, HPV type and HPV cofactors | Int J Cancer |
| Smith EM | 2007 | Human papillomavirus seropositivity and risks of head and neck cancer | Int J Cancer |
| Hassan MM | 2008 | Association between hepatitis B virus and pancreatic cancer | J Clin Oncol |
| Schöllkopf C | 2008 | Borrelia infection and risk of non-Hodgkin lymphoma | Blood |
| Cocco P | 2008 | Risk of malignant lymphoma following viral hepatitis infection | Int J Hematol |
| Shannon BA | 2008 | The antibody response to Propionibacterium acnes is an independent predictor of serum prostate-specific antigen levels in biopsy-negative men | BJU Int |
| Hsing AW | 2008 | Hepatitis B and C virus infection and the risk of biliary tract cancer: a population-based study in China | Int J Cancer |
| Tatemichi M | 2008 | Different etiological role of Helicobacter pylori (Hp) infection in carcinogenesis between differentiated and undifferentiated gastric cancers: a nested case-control study using IgG titer against Hp surface antigen | Acta Oncol |
| Adachi S | 2008 | Impact of occult hepatitis B virus infection and prior hepatitis B virus infection on development of hepatocellular carcinoma in patients with liver cirrhosis due to hepatitis C virus | Scand J Gastroenterol |
| Furniss CS | 2009 | Human papillomavirus 6 seropositivity is associated with risk of head and neck squamous cell carcinoma, independent of tobacco and alcohol use | Ann Oncol |
| Kim Y | 2009 | Epstein-Barr virus antibody level and gastric cancer risk in Korea: a nested case-control study | Br J Cancer |
| Jang JS | 2009 | Association of concurrent hepatitis B surface antigen and antibody to hepatitis B surface antigen with hepatocellular carcinoma in chronic hepatitis B virus infection | J Med Virol |
| Hsu WL | 2009 | Independent effect of EBV and cigarette smoking on nasopharyngeal carcinoma: a 20-year follow-up study on 9,622 males without family history in Taiwan | Cancer Epidemiol Biomarkers Prev |
| Rollison DE | 2009 | Prospective study of JC virus seroreactivity and the development of colorectal cancers and adenomas | Cancer Epidemiol Biomarkers Prev |
| Casabonne D | 2009 | A case-control study of cutaneous squamous cell carcinoma among Caucasian organ transplant recipients: the role of antibodies against human papillomavirus and other risk factors | Int J Cancer |
| Hishida A | 2010 | Smoking behavior and risk of Helicobacter pylori infection, gastric atrophy and gastric cancer in Japanese | Asian Pac J Cancer Prev |
| Chitose S | 2010 | Immunological responses against human papilloma virus and human papilloma virus induced laryngeal cancer | J Laryngol Otol |
| Cox B | 2010 | Breast cancer, cytomegalovirus and Epstein-Barr virus: a nested case-control study | Br J Cancer |
| Chen DY | 2010 | Polymyositis/dermatomyositis and nasopharyngeal carcinoma: the Epstein-Barr virus connection? | J Clin Virol |
| Severi G | 2010 | Plasma concentration of Propionibacterium acnes antibodies and prostate cancer risk: results from an Australian population-based case-control study | Br J Cancer |
| Bertrand KA | 2010 | A prospective study of Epstein-Barr virus antibodies and risk of non-Hodgkin lymphoma | Blood |
| Safaeian M | 2010 | Chlamydia trachomatis and risk of prevalent and incident cervical premalignancy in a population-based cohort | J Natl Cancer Inst |
| Chang KC | 2010 | Chronic hepatitis C increased the mortality rates of patients with hepatocellular carcinoma and diabetes mellitus in a triple hepatitis virus endemic community | J Gastroenterol |
| Wada Y | 2010 | Relationship between Helicobacter pylori tyrosine-phosphorylated CagA-related markers and the development of diffuse-type gastric cancers: a case-control study | Digestion |
| Persson C | 2011 | H. pylori seropositivity before age 40 and subsequent risk of stomach cancer: a glimpse of the true relationship? | PLoS One |
| Maricque BB | 2011 | Antibody responses to prostate-associated antigens in patients with prostatitis and prostate cancer | Prostate |
| Abnet CC | 2011 | Plasma pepsinogens, antibodies against Helicobacter pylori, and risk of gastric cancer in the Shanghai Women's Health Study Cohort | Br J Cancer |
| He JR | 2011 | Epstein-Barr virus and breast cancer: serological study in a high-incidence area of nasopharyngeal carcinoma | Cancer Lett |
| Cho EJ | 2011 | Relative etiological role of prior hepatitis B virus infection and nonalcoholic fatty liver disease in the development of non-B non-C hepatocellular carcinoma in a hepatitis B-endemic area | Digestion |
| Ribeiro KB | 2011 | Low human papillomavirus prevalence in head and neck cancer: results from two large case-control studies in high-incidence regions | Int J Epidemiol |
| Beachler DC | 2011 | Kaposi sarcoma-associated herpesvirus serum DNA and antibodies not associated with subsequent non-Hodgkin lymphoma risk | J Acquir Immune Defic Syndr |
| Agborsangaya CB | 2011 | Association between Epstein-Barr virus infection and risk for development of pregnancy-associated breast cancer: joint effect with vitamin D? | Eur J Cancer |
| Hsu WL | 2011 | Familial tendency and risk of nasopharyngeal carcinoma in taiwan: effects of covariates on risk | Am J Epidemiol |
| Cao SM | 2011 | Fluctuations of epstein-barr virus serological antibodies and risk for nasopharyngeal carcinoma: a prospective screening study with a 20-year follow-up | PLoS One |
| Khoshbaten M | 2011 | Helicobacter pylori infection reduces the risk of esophageal squamous cell carcinoma: a case-control study in iran | Asian Pac J Cancer Prev |
| Wang DS | 2012 | ABO blood group, hepatitis B viral infection and risk of pancreatic cancer | Int J Cancer |
| Andersson K | 2012 | Prospective study of human papillomavirus seropositivity and risk of nonmelanoma skin cancer | Am J Epidemiol |
| Ben Q | 2012 | Hepatitis B virus status and risk of pancreatic ductal adenocarcinoma: a case-control study from China | Pancreas |
| Ahn J | 2012 | Periodontal disease, Porphyromonas gingivalis serum antibody levels and orodigestive cancer mortality | Carcinogenesis |
| Epplein M | 2012 | Prospective study of Helicobacter pylori biomarkers for gastric cancer risk among Chinese men | Cancer Epidemiol Biomarkers Prev |
| Mueller NE | 2012 | Antibody titers against EBNA1 and EBNA2 in relation to Hodgkin lymphoma and history of infectious mononucleosis | Int J Cancer |
| Watanabe M | 2012 | Development of gastric cancer in nonatrophic stomach with highly active inflammation identified by serum levels of pepsinogen and Helicobacter pylori antibody together with endoscopic rugal hyperplastic gastritis | Int J Cancer |
| Robles C | 2013 | Bladder cancer and seroreactivity to BK, JC and Merkel cell polyomaviruses: the Spanish bladder cancer study | Int J Cancer |
| Michaud DS | 2013 | Plasma antibodies to oral bacteria and risk of pancreatic cancer in a large European prospective cohort study | Gut |
| Epplein M | 2013 | Helicobacter pylori protein-specific antibodies and risk of colorectal cancer | Cancer Epidemiol Biomarkers Prev |
| Andersson K | 2013 | Prospective study of genital human papillomaviruses and nonmelanoma skin cancer | Int J Cancer |
| Karami N | 2013 | Seroreactivity to Helicobacter pylori antigens as a risk indicator of gastric cancer | Asian Pac J Cancer Prev |
| Anantharaman D | 2013 | Human papillomavirus infections and upper aero-digestive tract cancers: the ARCAGE study | J Natl Cancer Inst |
| Xing S | 2013 | Chronic hepatitis virus infection increases the risk of pancreatic cancer: a meta-analysis | Hepatobiliary Pancreat Dis Int |
| Reddy A | 2013 | Latent hepatitis B is a risk factor for hepatocellular carcinoma in patients with chronic hepatitis C | World J Gastroenterol |
| Risch HA | 2014 | Helicobacter pylori seropositivities and risk of pancreatic carcinoma | Cancer Epidemiol Biomarkers Prev |
| He Z | 2014 | Anti-HPV-E7 seropositivity and risk of esophageal squamous cell carcinoma in a high-risk population in China | Carcinogenesis |
| Liu J | 2014 | Serum Helicobacter pylori NapA antibody as a potential biomarker for gastric cancer | Sci Rep |
| Seo SI | 2014 | Coexistence of hepatitis B surface antigen and antibody to hepatitis B surface may increase the risk of hepatocellular carcinoma in chronic hepatitis B virus infection: a retrospective cohort study | J Med Virol |
| Coghill AE | 2014 | Epstein-Barr virus serology as a potential screening marker for nasopharyngeal carcinoma among high-risk individuals from multiplex families in Taiwan | Cancer Epidemiol Biomarkers Prev |
| Combes JD | 2014 | Antibodies against high-risk human papillomavirus proteins as markers for invasive cervical cancer | Int J Cancer |
| Faust H | 2014 | Prospective study of Merkel cell polyomavirus and risk of Merkel cell carcinoma | Int J Cancer |
| Anantharaman D | 2014 | No causal association identified for human papillomavirus infections in lung cancer | Cancer Res |
| Chang CM | 2014 | GBV-C infection and risk of NHL among U.S. adults | Cancer Res |
| Tian W | 2014 | Serum antibody against Helicobacter pylori FlaA and risk of gastric cancer | Helicobacter |
| Castellsagué X | 2014 | Prospective seroepidemiologic study on the role of Human Papillomavirus and other infections in cervical carcinogenesis: evidence from the EPIC cohort | Int J Cancer |
| Song H | 2014 | A CagA-independent cluster of antigens related to the risk of noncardia gastric cancer: associations between Helicobacter pylori antibodies and gastric adenocarcinoma explored by multiplex serology | Int J Cancer |
| Michaud DS | 2014 | High-risk HPV types and head and neck cancer | Int J Cancer |
| Al-Kubaisy WA | 2014 | Hepatitis C virus prevalence and genotyping among hepatocellular carcinoma patients in Baghdad | Asian Pac J Cancer Prev |
| Castellsagué X | 2014 | Risk of newly detected infections and cervical abnormalities in women seropositive for naturally acquired human papillomavirus type 16/18 antibodies: analysis of the control arm of PATRICIA | J Infect Dis |
| Lee ST | 2014 | Interaction of allergy history and antibodies to specific varicella-zoster virus proteins on glioma risk | Int J Cancer |
| Yoshida T | 2014 | Cancer development based on chronic active gastritis and resulting gastric atrophy as assessed by serum levels of pepsinogen and Helicobacter pylori antibody titer | Int J Cancer |
| Lang Kuhs KA | 2015 | Human Papillomavirus 16 E6 Antibodies in Individuals without Diagnosed Cancer: A Pooled Analysis | Cancer Epidemiol Biomarkers Prev |
| Teras LR | 2015 | Prediagnostic circulating polyomavirus antibody levels and risk of non-Hodgkin lymphoma | Cancer Epidemiol Biomarkers Prev |
| Colombara DV | 2015 | Prior human polyomavirus and papillomavirus infection and incident lung cancer: a nested case-control study | Cancer Causes Control |
| Anderson KS | 2015 | HPV16 antibodies as risk factors for oropharyngeal cancer and their association with tumor HPV and smoking status | Oral Oncol |
| Gonzalez HC | 2015 | Chronic hepatitis C infection as a risk factor for renal cell carcinoma | Dig Dis Sci |
| Kreimer AR | 2015 | Human papillomavirus antibodies and future risk of anogenital cancer: a nested case-control study in the European prospective investigation into cancer and nutrition study | J Clin Oncol |
| Cárdenas-Mondragón MG | 2015 | Case–control study of Epstein–Barr virus and Helicobacter pylori serology in Latin American patients with gastric disease | Br J Cancer |
| Apter D | 2015 | Efficacy of human papillomavirus 16 and 18 (HPV-16/18) AS04-adjuvanted vaccine against cervical infection and precancer in young women: final event-driven analysis of the randomized, double-blind PATRICIA trial | Clin Vaccine Immunol |
| Blase JL | 2016 | Prediagnostic Helicobacter pylori Antibodies and Colorectal Cancer Risk in an Elderly, Caucasian Population | Helicobacter |
| Kerishnan JP | 2016 | Detection of Human Papillomavirus 16-Specific IgG and IgM Antibodies in Patient Sera: A Potential Indicator of Oral Squamous Cell Carcinoma Risk Factor | Int J Med Sci |
| Li H | 2016 | Serum Helicobacter pylori FliD antibody and the risk of gastric cancer | Oncotarget |
| Amougou MA | 2016 | A prominent role of Hepatitis D Virus in liver cancers documented in Central Africa | BMC Infect Dis |
| Coghill AE | 2016 | High Levels of Antibody that Neutralize B-cell Infection of Epstein-Barr Virus and that Bind EBV gp350 Are Associated with a Lower Risk of Nasopharyngeal Carcinoma | Clin Cancer Res |
| Cai H | 2016 | Helicobacter pylori blood biomarker for gastric cancer risk in East Asia | Int J Epidemiol |
| Kayamba V | 2016 | Serological response to Epstein-Barr virus early antigen is associated with gastric cancer and human immunodeficiency virus infection in Zambian adults: a case-control study | Pan Afr Med J |
| Anderson KS | 2017 | Pre-diagnostic dynamic HPV16 IgG seropositivity and risk of oropharyngeal cancer | Oral Oncol |
| Kreimer AR | 2017 | Kinetics of the Human Papillomavirus Type 16 E6 Antibody Response Prior to Oropharyngeal Cancer | J Natl Cancer Inst |
| Marous M | 2017 | Trichomonas vaginalis infection and risk of prostate cancer: associations by disease aggressiveness and race/ethnicity in the PLCO Trial | Cancer Causes Control |
| Wang Z | 2018 | Role of serum EBV-VCA IgG detection in assessing gastric cancer risk and prognosis in Northern Chinese population | Cancer Med |
| Shepherd L | 2018 | The extent of B-cell activation and dysfunction preceding lymphoma development in HIV-positive people | HIV Med |
| Butt J | 2018 | Prospective evaluation of antibody response to Streptococcus gallolyticus and risk of colorectal cancer | Int J Cancer |
| Bassig BA | 2018 | Serologic markers of viral infection and risk of non-Hodgkin lymphoma: A pooled study of three prospective cohorts in China and Singapore | Int J Cancer |
| Varga MG | 2018 | Epstein-Barr Virus Antibody Titers Are Not Associated with Gastric Cancer Risk in East Asia | Dig Dis Sci |
| Coghill AE | 2018 | Evaluation of Total and IgA-Specific Antibody Targeting Epstein-Barr Virus Glycoprotein 350 and Nasopharyngeal Carcinoma Risk | J Infect Dis |
| Liu X | 2018 | Interaction between tobacco smoking and hepatitis B virus infection on the risk of liver cancer in a Chinese population | Int J Cancer |
| Varga MG | 2018 | Helicobacter pylori Blood Biomarkers and Gastric Cancer Survival in China | Cancer Epidemiol Biomarkers Prev |
| Trabert B | 2019 | Antibodies Against Chlamydia trachomatis and Ovarian Cancer Risk in Two Independent Populations | J Natl Cancer Inst |
| Aragonés N | 2019 | Epstein Barr virus antibody reactivity and gastric cancer: A population-based case-control study | Cancer Epidemiol |
| Kreimer AR | 2019 | Timing of HPV16-E6 antibody seroconversion before OPSCC: findings from the HPVC3 consortium | Ann Oncol |
| Klatka J | 2019 | T-Lymphocyte Activation Is Correlated With the Presence of Anti-EBV in Patients With Laryngeal Squamous Cell Carcinoma | In Vivo |
| Rajendra S | 2020 | Antibodies against human papillomavirus proteins in Barrett's dysplasia and intramucosal esophageal adenocarcinoma | Ann N Y Acad Sci |
| Lv YL | 2020 | Cytomegalovirus Infection Is a Risk Factor in Gastrointestinal Cancer: A Cross-Sectional and Meta-Analysis Study | Intervirology |
| Idahl A | 2020 | Serologic markers of Chlamydia trachomatis and other sexually transmitted infections and subsequent ovarian cancer risk: Results from the EPIC cohort | Int J Cancer |
| Komori MF | 2020 | Epidemiological Correlations Between Head and Neck Cancer and Hepatitis B Core Antibody Positivity | Anticancer Res |
| Inoue M | 2020 | High-Negative Anti-Helicobacter pylori IgG Antibody Titers and Long-Term Risk of Gastric Cancer: Results from a Large-Scale Population-Based Cohort Study in Japan | Cancer Epidemiol Biomarkers Prev |
| Fernandez-Botran R | 2020 | Seroprevalence of Helicobacter pylori/CagA Antibodies in Guatemalan Gastric Cancer Patients: Association of Seropositivity with Increased Plasma Levels of Pepsinogens but not Soluble Urokinase Plasminogen Activator Receptor | Am J Trop Med Hyg |
| Gareayaghi N | 2021 | Epstein-Barr Virus and Helicobacter pylori co-infection in patients with gastric cancer and duodenale ulcer | New Microbiol |
| Saleh NE | 2021 | Trichomonas vaginalis serostatus and prostate cancer risk in Egypt: a case-control study | Parasitol Res |
| Amorrortu RP | 2021 | Association between Human Polyomaviruses and Keratinocyte Carcinomas: A Prospective Cohort Study | Cancer Epidemiol Biomarkers Prev |
| Bettampadi D | 2021 | Factors Associated With Persistence and Clearance of High-Risk Oral Human Papillomavirus (HPV) Among Participants in the HPV Infection in Men (HIM) Study | Clin Infect Dis |
| Self-reactive antibodies | | | |
| Shimoyama T | 2000 | Serum anti-Lewis X antibody is not elevated in patients with gastric cancer infected with Helicobacter pylori | J Clin Gastroenterol |
| Ralhan R | 2000 | Circulating p53 antibodies, p53 gene mutational profile and product accumulation in esophageal squamous-cell carcinoma in India | Int J Cancer |
| Hahn JS | 2001 | Non-Hodgkin's lymphoma & primary biliary cirrhosis with Sjögren's syndrome | Yonsei Med J |
| Le Naour F | 2002 | A distinct repertoire of autoantibodies in hepatocellular carcinoma identified by proteomic analysis | Mol Cell Proteomics |
| Neri M | 2003 | Serum anti-p53 autoantibodies in pleural malignant mesothelioma, lung cancer and non-neoplastic lung diseases | Lung Cancer |
| Boi F | 2005 | High prevalence of suspicious cytology in thyroid nodules associated with positive thyroid autoantibodies | Eur J Endocrinol |
| Cramer DW | 2005 | Conditions associated with antibodies against the tumor-associated antigen MUC1 and their relationship to risk for ovarian cancer | Cancer Epidemiol Biomarkers Prev |
| Zhong L | 2006 | Profiling tumor-associated antibodies for early detection of non-small cell lung cancer | J Thorac Oncol |
| Malik M | 2007 | Relationship between cancer and oral pemphigoid patients with antibodies to alpha6-integrin | J Oral Pathol Med |
| Yamazawa K | 2007 | Serum p53 antibody as a diagnostic marker of high-risk endometrial cancer | Am J Obstet Gynecol |
| Hermsen BB | 2007 | Humoral immune responses to MUC1 in women with a BRCA1 or BRCA2 mutation | Eur J Cancer |
| Yoshizawa S | 2007 | Clinical significance of serum p53 antibodies in patients with ulcerative colitis and its carcinogenesis | Inflamm Bowel Dis |
| Pontifex EK | 2007 | Risk factors for lung cancer in patients with scleroderma: a nested case-control study | Ann Rheum Dis |
| Chapman CJ | 2008 | Autoantibodies in lung cancer: possibilities for early detection and subsequent cure | Thorax |
| Malhotra RK | 2008 | Poorly differentiated gastroenteropancreatic neuroendocrine carcinoma associated with X-linked hyperimmunoglobulin M syndrome | Arch Pathol Lab Med |
| Bazzan M | 2009 | Presence of low titre of antiphospholipid antibodies in cancer patients: a prospective study | Intern Emerg Med |
| Fiore E | 2009 | Lower levels of TSH are associated with a lower risk of papillary thyroid cancer in patients with thyroid nodular disease: thyroid autonomy may play a protective role | Endocr Relat Cancer |
| Kim ES | 2010 | Thyroglobulin antibody is associated with increased cancer risk in thyroid nodules | Thyroid |
| Wu M | 2010 | Serum p53 protein and anti-p53 antibodies are associated with increased cancer risk: a case-control study of 569 patients and 879 healthy controls | Mol Biol Rep |
| Yasuda M | 2010 | Identification of a tumour associated antigen in lung cancer patients with asbestos exposure | Anticancer Res |
| Gulati D | 2010 | Primary Sjogren's syndrome with anticentromere antibodies--a clinically distinct subset | Clin Rheumatol |
| Azizi G | 2011 | Autoimmune thyroid disease: a risk factor for thyroid cancer | Endocr Pract |
| Koshiol J | 2011 | Racial differences in chronic immune stimulatory conditions and risk of non-Hodgkin's lymphoma in veterans from the United States | J Clin Oncol |
| Li Y | 2012 | Elevated serum antibodies against insulin-like growth factor-binding protein-2 allow detecting early-stage cancers: evidences from glioma and colorectal carcinoma studies | Ann Oncol |
| Böckle BC | 2012 | Analysis of 303 Ro/SS-A antibody-positive patients: is this antibody a possible marker for malignancy? | Br J Dermatol |
| Tosovic A | 2012 | Prospectively measured thyroid hormones and thyroid peroxidase antibodies in relation to breast cancer risk | Int J Cancer |
| Zhang X | 2013 | Identification of novel autoantibodies for detection of malignant mesothelioma | PLoS One |
| Pedersen JW | 2013 | Early detection of cancer in the general population: a blinded case-control study of p53 autoantibodies in colorectal cancer | Br J Cancer |
| Hellstrom I | 2013 | Anti-HE4 antibodies in infertile women and women with ovarian cancer | Gynecol Oncol |
| Lun Y | 2013 | Hashimoto's thyroiditis as a risk factor of papillary thyroid cancer may improve cancer prognosis | Otolaryngol Head Neck Surg |
| Lowe FJ | 2014 | A novel autoantibody test for the detection of pre-neoplastic lung lesions | Mol Cancer |
| Moinzadeh P | 2014 | Association of anti-RNA polymerase III autoantibodies and cancer in scleroderma | Arthritis Res Ther |
| Cho YA | 2014 | Biomarkers of thyroid function and autoimmunity for predicting high-risk groups of thyroid cancer: a nested case-control study | BMC Cancer |
| Vasileiadis I | 2014 | Thyroglobulin antibodies could be a potential predictive marker for papillary thyroid carcinoma | Ann Surg Oncol |
| Jia J | 2014 | Development of a multiplex autoantibody test for detection of lung cancer | PLoS One |
| Middleton CH | 2014 | Serum autoantibody measurement for the detection of hepatocellular carcinoma | PLoS One |
| Wu X | 2014 | Coexistence of thyroglobulin antibodies and thyroid peroxidase antibodies correlates with elevated thyroid-stimulating hormone level and advanced tumor stage of papillary thyroid cancer | Endocrine |
| Zhou SL | 2014 | Autoantibody detection to tumor-associated antigens of P53, IMP1, P16, cyclin B1, P62, C-myc, Survivn, and Koc for the screening of high-risk subjects and early detection of esophageal squamous cell carcinoma | Dis Esophagus |
| Shah AA | 2015 | Examination of autoantibody status and clinical features associated with cancer risk and cancer-associated scleroderma | Arthritis Rheumatol |
| Wong SL | 2015 | Anti-thyroid antibodies as a predictor of thyroid cancer | ANZ J Surg |
| Qin J | 2015 | High Thyroglobulin Antibody Levels Increase the Risk of Differentiated Thyroid Carcinoma | Dis Markers |
| Molina-Ruiz AM | 2015 | Amyophatic dermatomyositis presenting as a flagellated skin eruption with positive MDA5 antibodies and thyroid cancer: a real association? | Clin Exp Dermatol |
| Grani G | 2015 | Thyroid autoimmunity and risk of malignancy in thyroid nodules submitted to fine-needle aspiration cytology | Head Neck |
| Sun Y | 2016 | A prospective study of autoantibodies to Ezrin and pancreatic cancer risk | Cancer Causes Control |
| Allenbach Y | 2016 | High risk of cancer in autoimmune necrotizing myopathies: usefulness of myositis specific antibody | Brain |
| Yu Y | 2016 | Clinical Relationship Between IgG4-Positive Hashimoto's Thyroiditis and Papillary Thyroid Carcinoma | J Clin Endocrinol Metab |
| Lohmueller JJ | 2016 | Antibodies elicited by the first non-viral prophylactic cancer vaccine show tumor-specificity and immunotherapeutic potential | Sci Rep |
| Hosseini S | 2016 | Can preoperative thyroglobulin antibody levels be used as a marker for well differentiated thyroid cancer? | J Otolaryngol Head Neck Surg |
| Szender JB | 2016 | HLA superfamily assignment is a predictor of immune response to cancer testis antigens and survival in ovarian cancer | Gynecol Oncol |
| Selek A | 2017 | Thyroid autoimmunity: is really associated with papillary thyroid carcinoma? | Eur Arch Otorhinolaryngol |
| Pilyugin M | 2017 | BARD1 serum autoantibodies for the detection of lung cancer | PLoS One |
| Yang H | 2017 | Identification of multiple cancer-associated myositis-specific autoantibodies in idiopathic inflammatory myopathies: a large longitudinal cohort study | Arthritis Res Ther |
| Ceribelli A | 2017 | Myositis-specific autoantibodies and their association with malignancy in Italian patients with polymyositis and dermatomyositis | Clin Rheumatol |
| Lazzaroni MG | 2017 | Malignancies in Patients with Anti-RNA Polymerase III Antibodies and Systemic Sclerosis: Analysis of the EULAR Scleroderma Trials and Research Cohort and Possible Recommendations for Screening | J Rheumatol |
| Shah AA | 2017 | Brief Report: Anti-RNPC-3 Antibodies As a Marker of Cancer-Associated Scleroderma | Arthritis Rheumatol |
| Yu-Rice Y | 2017 | Selenium-Binding Protein 1 (SBP1) autoantibodies in ovarian disorders and ovarian cancer | Reproduction |
| Banjara M | 2017 | Detection of brain-directed autoantibodies in the serum of non-small cell lung cancer patients | PLoS One |
| Teras LR | 2018 | Prediagnostic Antibodies to Serum p53 and Subsequent Colorectal Cancer | Cancer Epidemiol Biomarkers Prev |
| Cramer DW | 2018 | Anti-CA15.3 and Anti-CA125 Antibodies and Ovarian Cancer Risk: Results from the EPIC Cohort | Cancer Epidemiol Biomarkers Prev |
| Bost C | 2018 | Malignant tumors in autoimmune encephalitis with anti-NMDA receptor antibodies | J Neurol |
| Kimman J | 2018 | Prognostic value of cryoglobulins, protein electrophoresis, and serum immunoglobulins for lymphoma development in patients with Sjögren's syndrome. A retrospective cohort study | Acta Clin Belg |
| Yoo J | 2018 | Cancer development in Korean patients with ANCA-associated vasculitis: a single centre study | Clin Exp Rheumatol |
| Betteridge ZE | 2018 | Investigation of myositis and scleroderma specific autoantibodies in patients with lung cancer | Arthritis Res Ther |
| Igusa T | 2018 | Autoantibodies and scleroderma phenotype define subgroups at high-risk and low-risk for cancer | Ann Rheum Dis |
| Samraj AN | 2018 | Polyclonal human antibodies against glycans bearing red meat-derived non-human sialic acid N-glycolylneuraminic acid are stable, reproducible, complex and vary between individuals: Total antibody levels are associated with colorectal cancer risk | PLoS One |
| Baecklund E | 2018 | Anti-cyclic citrullinated peptide antibodies, other common autoantibodies, and smoking as risk factors for lymphoma in patients with rheumatoid arthritis | Scand J Rheumatol |
| Zhao H | 2018 | High Urinary Iodine, Thyroid Autoantibodies, and Thyroid-Stimulating Hormone for Papillary Thyroid Cancer Risk | Biol Trace Elem Res |
| Zhang X | 2018 | Correlation analyses of thyroid-stimulating hormone and thyroid autoantibodies with diﬀerentiated thyroid cancer | J BUON |
| Baldini C | 2018 | Artificial neural networks help to identify disease subsets and to predict lymphoma in primary Sjögren's syndrome | Clin Exp Rheumatol |
| Zhao H | 2018 | Alteration of circulating natural autoantibodies to CD25-derived peptide antigens and FOXP3 in non-small cell lung cancer | Sci Rep |
| Shah AA | 2019 | Protective Effect Against Cancer of Antibodies to the Large Subunits of Both RNA Polymerases I and III in Scleroderma | Arthritis Rheumatol |
| Guo X | 2019 | Hyperinsulinemia and thyroid peroxidase antibody in Chinese patients with papillary thyroid cancer | Endocr J |
| Huo Y | 2020 | Case study of an autoantibody panel for early detection of lung cancer and ground-glass nodules | J Cancer Res Clin Oncol |
| Morrisroe K | 2020 | Incidence, Risk Factors, and Outcomes of Cancer in Systemic Sclerosis | Arthritis Care Res (Hoboken) |
| Adhami M | 2020 | Anti-Thyroid Antibodies and TSH as Potential Markers of Thyroid Carcinoma and Aggressive Behavior in Patients with Indeterminate Fine-Needle Aspiration Cytology | World J Surg |
| Kårhus LL | 2020 | Long-term Consequences of Undiagnosed Celiac Seropositivity | Am J Gastroenterol |
| Mofors J | 2020 | Increased risk of multiple myeloma in primary Sjögren's syndrome is limited to individuals with Ro/SSA and La/SSB autoantibodies | Ann Rheum Dis |
| Li SY | 2020 | Association between serum anti‑ASXL2 antibody levels and acute ischemic stroke, acute myocardial infarction, diabetes mellitus, chronic kidney disease and digestive organ cancer, and their possible association with atherosclerosis and hypertension | Int J Mol Med |
| Wang M | 2021 | Tumor-associated autoantibodies in ESCC screening: Detecting prevalent early-stage malignancy or predicting future cancer risk? | EBioMedicine |
| Leivo MZ | 2021 | Expression of uroplakin II and GATA-3 in bladder cancer mimickers: caveats in the use of a limited panel to determine cell of origin in bladder lesions | Hum Pathol |
